# Sensor Fusion with Asynchronous Decentralized Processing for 3D Target Tracking with a Wireless Camera Network

**DOI:** 10.3390/s23031194

**Published:** 2023-01-20

**Authors:** Thiago Marchi Di Gennaro, Jacques Waldmann

**Affiliations:** 1 Systems and Control Department, Weapons Systems Directorate, Brazilian Navy, Rio de Janeiro 20010-100, RJ, Brazil; 2 Systems and Control Department, Eletronics Engineering Division, Instituto Tecnológico de Aeronáutica, São José dos Campos 12228-900, SP, Brazil

**Keywords:** 3D tracking, line-of-sight fusion, camera network, processing delay, asynchrony

## Abstract

We present a method to acquire 3D position measurements for decentralized target tracking with an asynchronous camera network. Cameras with known poses have fields of view with overlapping projections on the ground and 3D volumes above a reference ground plane. The purpose is to track targets in 3D space without constraining motion to a reference ground plane. Cameras exchange line-of-sight vectors and respective time tags asynchronously. From stereoscopy, we obtain the fused 3D measurement at the local frame capture instant. We use local decentralized Kalman information filtering and particle filtering for target state estimation to test our approach with only local estimation. Monte Carlo simulation includes communication losses due to frame processing delays. We measure performance with the average root mean square error of 3D position estimates projected on the image planes of the cameras. We then compare only local estimation to exchanging additional asynchronous communications using the Batch Asynchronous Filter and the Sequential Asynchronous Particle Filter for further fusion of information pairs’ estimates and fused 3D position measurements, respectively. Similar performance occurs in spite of the additional communication load relative to our local estimation approach, which exchanges just line-of-sight vectors.

## 1. Introduction

Tracking multiple targets in a distributed-processing wireless-communication network (WCN) of cameras encompasses several areas such as surveillance, computer vision, threat detection [1], inertial navigation aided by additional sensors, and autonomous vehicles. In the tracking process investigated here, we assume fixed cameras whose poses are known and with computational processing resources (network nodes). The cameras estimate the location of a moving object of interest, here called a target, over time [2] in three-dimensional (3D) space. Network topology affects the distributed processing that aims at greater flexibility and robustness [3], and target tracking is expected to become less likely subject to failure. Each network node implements detection algorithms and state estimators, where state estimate refers to the position and velocity of the estimated target. Detected targets are represented by the location of their centroids on the image plane, and the corresponding measurements are the line-of-sight (*LoS*) vectors. Target 3D position arises from intersecting *LoS* vectors measured by neighboring nodes covering the 3D space the target traverses. Hence, the target 3D motion is not restricted to a reference ground plane, and targets can move freely in the 3D space covered by the field of view of each camera in the network. The *LoS* measurements are usually noisy or erroneous owing to the limitations of computer vision algorithms [4]. With a network of cameras, it is possible to improve target state estimation via fusion of information from different cameras observing the same target.

Two approaches for information exchange among cameras are found in the literature: measurement information exchange [3,5,6] and state estimate information exchange [7,8,9,10,11,12,13,14,15,16,17,18]. The former computes the fusion of exchanged measurements and subsequently proceeds to target state estimation. Concerning the target state estimate exchange, the *i*-th camera fuses the local state estimate with the same target’s state estimate received from neighboring cameras.

Each camera node has a local clock representing the timeline for each of its pre-programmed actions. The network nodes share the same inter-frame interval, though frame capture instants are asynchronous [8]. Moreover, for each captured frame, there is a processing delay when detecting the targets and computing the measurements. Here, this measurement is the 3D target position in world Cartesian coordinates from intersecting local and received *LoS* vectors of cameras with known poses followed by the unscented transform (UT) [19]. Processing delay and asynchronous capture are both integer multiples of the inter-frame interval and dealt with by means of temporal alignment of the 3D position measurements with the local frame capture instant and measurement fusion by averaging the aligned 3D position measurements. Then, we proceed with the local estimation of either the target information pair with Kalman filtering in information form or the target state estimate with particle filtering.

We compare the above with the Batch Asynchronous Filter (BAF) [15] and the Sequential Asynchronous Particle Filter (SA-PF) [17], both Bayesian decentralized filters, in combination with our fused 3D position measurement approach. BAF and SA-PF originally exchange information other than just asynchronous line-of-sight vectors to targets, respectively, asynchronous information pairs’ estimates, and fused 3D position measurements.

BAF exchanges asynchronous information pair estimates among neighboring cameras from local Kalman information filters, aligns them temporally at the frame capture instant for Kullback–Leibler Average (KLA) fusion [20], and proceeds to target information pair update followed by time propagation to the next local capture instant.

SA-PF [17] asynchronously exchanges fused 3D position measurements among neighboring cameras, then propagates each local particle state estimate to the reception instant of each measurement in the local reception sequence, iteratively updates each local particle weight with the likelihood of each received measurement, updates the local state estimate, and propagates to the next local capture instant.

### 1.1. Related Work

Target tracking in a distributed asynchronous camera network can either use a sequential method [13] or a batch method [6]. In sequential methods, a network node computes the target state not only when local measurements are obtained but also when the same node receives information from neighboring camera nodes. The nodes do not propagate the estimates received from neighboring nodes to alignment with the local frame capture instant prior to update; the reception instants are used as the sending camera frame capture instants and the probability density for target state estimation; namely, the particle weights are updated sequentially [15,17]. The SA-PF filter disregards the processing delays.

In batch processing methods, the update with data information received asynchronously from other nodes occurs after the received data are propagated to the local frame capture instant for the purpose of time alignment [6,15]. BAF [15] considers the asynchronous frame capturing among cameras and the processing delay in the estimation and fusion phases at each camera. In the estimation phase, a camera obtains the 3D position measurement assuming the target motion develops on a ground reference plane and estimates the information pair at the frame capture instant. Cameras exchange local target state information, and all information received during a time window period is stored in a buffer. At the end of the time window, a camera enters the fusion phase and propagates the received target information pairs of other cameras either backward or forward to temporal alignment with the local frame capture instant [21].

Previous work mostly related to target tracking with a camera network used information exchange of two-dimensional target states restricted to move on a reference ground plane [2,3,7,8,10,13,14,15,17,21]. However, much less attention has been given to the often-occurring scenario in which targets move in 3D space while taking into account network asynchrony and delays in image processing. For example, in [22], multi-view histograms were employed to characterize targets in 3D space using color and texture as the visual features; the implementation was based on particle filtering. In [23], a deep network model was trained with an RGB-D dataset, and then the 3D structure of the target was reconstructed from the corresponding RGB image to predict depth information. In [24], images with a detected hand and head were used with each camera transmitting to a central processor using a low-power communication network. The central processor performed 3D pose reconstruction combining the data in a probabilistic manner. In [16], state estimation with complete maximum likelihood information transfer produced 3D cartesian estimates of target position and velocity from azimuth and elevation provided by multiple cameras. Ref. [25] addressed the competing objectives of monitoring 3D indoor structured areas against tracking multiple 3D points with a heterogeneous visual sensor network composed of both fixed and Pan-Tilt-Zoom (PTZ) controllable cameras. The measurement was the projection of a distinct 3D point representing the target onto an image plane wherein all cameras followed the pinhole model with unit focal length. The authors applied decentralized EKF information filtering, neither handling camera network asynchrony nor the issue that in real image sequences a target occupies a volume; thus, cameras aiming at a target from different directions see quite distinct portions of the target surface and different 3D points.

None of the above methods considered frame processing delays and network asynchrony among camera nodes.

Reference [26] applied DAT2TF to an asynchronous network of 2D radars that provided local Kalman filter state estimates to a central fusion node performing global estimation of the 3D target state. While focusing on the fusion of state estimates, a linearized approach is highlighted to determine the Cramer–Rao estimation error uncertainty bound when converting radar measurements from polar to Cartesian coordinates, and the authors claim the result is equivalent to decentralized filtering.

### 1.2. Our Proposal

To track multiple actual targets undergoing unrestricted motion in a real image sequence with a network of calibrated cameras whose poses are known, we propose the decentralized approach wherein our focus is on the fusion of 3D position measurements combined with only local estimation, without involvement of a central node. For that, we need to invert the projection function from 3D space to the image plane with stereoscopy and the known camera poses and calibration data:

Step 1: Within a time window about the local frame capture instant and among neighboring cameras, transmit and receive asynchronously *LoS* vectors represented in world coordinate system {W} to a target centroid in the image plane and the corresponding time stamps among neighboring cameras within a time window about the frame capture instant [15];

Step 2: Use the unscented transform (UT) and stereovision principles to acquire 3D position measurements and covariances from the received *LoS* vectors when they intersect to within a 3D spatial distance threshold;

Step 3: Propagate forward or backward in time the 3D position measurements and respective covariances via interpolation until temporal alignment with the local frame capture instant;

Step 4: Fuse locally the 3D position measurements and covariances at the frame capture instant by means of averaging, use them to update the local estimate, and propagate to the next frame capture instant.

We compare our approach to SA-PF and BAF wherein additional communication is exchanged other than just *LoS* vectors to measure 3D positions. After the aforementioned time window in Step 1, each camera starts a fusion window after the time window to receive and process the additional communications. In SA-PF, a camera transmits and receives fused 3D position measurements and respective covariances at distinct instants in the fusion window. Differently, BAF receives asynchronous information pairs’ estimates at distinct instants in the fusion window.

Both BAF and SA-PF are compared to our proposal with the average root mean square error in the image planes of the cameras tracking the various targets in the scene. The comparison also aims at investigating whether there is a relevant change in performance when a camera fails to receive communications due to ongoing frame processing.

### 1.3. Contributions

Our innovative approach directly measures the 3D centroid position of an actual target with stereoscopy as different, calibrated cameras with known poses view distinct portions of the surface of the same target. Moreover, we explicitly dispense with the fusion node to add robustness to target tracking via decentralized processing in a camera network with partial connectivity. Our focus is on the fusion of 3D position measurements and respective covariances computed with *LoS* vectors received asynchronously from neighboring cameras. We also consider the image processing delay of each node when obtaining the local measurements.

We derive *LoS* vectors from the optical center of each camera to the various target centroids in the respective image plane with the corresponding error covariances. Then, we use stereoscopy combined with an unscented transform (UT) that employs the minimum distance search between pairs of *LoS* vectors to compute the corresponding 3D positions and error covariance in the world reference frame. One *LoS* vector is from the local camera and the other *LoS* vector is received asynchronously from any of the neighboring cameras tracking the same target. We handle asynchronous *LoS* vectors and the corresponding error covariances, all represented in the world coordinate frame, with time propagation from the received instant into alignment with the camera local capture instant. A set of 3D position measurements and covariances arise with representation in the world coordinate frame at the capture instant of the camera. Then, we fuse the measurements and covariances by means of averaging and proceed to local estimation.

We emphasize that our contribution is not in filtering, though we modified both BAF and SA-PF to add a fusion stage to test our fused 3D position measurement approach and local estimation against a further fusion step among the neighboring cameras, and thus investigate the tradeoff between performance and required communications. We find that local estimation provides similar performance with a smaller communication workload. 

Moreover, in the real scenario evaluated here, namely the PETS2009 sequence [27], subjects enter and leave the fields of view of cameras the camera network, resulting in targets that merge and separate as they suddenly change motion direction in 3D space. Our approach handles such occurrences gracefully. According to our research, no previous investigation has focused on decentralized target tracking using *LoS* vectors exchanged asynchronously among neighboring camera nodes with frame processing delay to obtain position measurements in 3D space from stereoscopy principles. In Table 1, we address an analytical comparison of methods found in the literature. 

### 1.4. Paper Structure

The paper is organized as follows. Section 2 formulates how to measure 3D position considering the processing delay, network asynchrony, and tracking without constraining target motion to a reference ground plane. Section 3 describes the algorithms to obtain 3D measurements and the modifications to the Bayesian BAF and SA-PF filter algorithms to include an additional fusion window for comparison with just local estimation. Section 4 presents simulation results and discusses performance, and Section 5 concludes the paper.

## 2. Problem Formulation

Some background is required initially:

- Variable *k* instants or time steps within a time window: they are integer multiples of the camera inter-frame interval and represent the capture instants of target centroids, and the instants of *LoS* vectors are received from neighboring cameras during the time window about the frame capture instant $k_{c}$ of a local camera. They are stored in the *i*-th camera node;
- Frame capture instants $k_{c}$: the moment when a frame is captured and defines a time window;
- Here we assume that the communication is ideal among cameras, i.e., there is no communication delays.

Unlike the fully connected network in [15,17], we investigate a partially connected network. Consider a WCN of cameras $C^{i}$, $i=\left\{1,2,\ldots ,N_{s}\right\}$, where $N_{s}$ denotes the maximum number of sensors. This network is based on decentralized processing, meaning that each $C^{i}$ camera node fuses its information with the information received from the set $N^{j,i}$ of camera nodes $C^{j},j\neq i$ considered neighbors; i.e., $N^{j,i}$ is the set of cameras with which the *i*-th camera communicates to track targets in 3D space. The network topology is fixed and independent of the tracked target. The term decentralized indicates that each camera in the network has a communication module, with detection and tracking algorithms independent from the rest of the cameras. There is no fusion center involved. Given that we aim at 3D tracking, the state of the *t*-th target at the *i*-th camera is given by $x_{t,k_{c},3D}^{i}={\left[x_{t,k_{c}}^{i},{\dot{x}}_{t,k_{c}}^{i},y_{t,k_{c}}^{i},{\dot{y}}_{t,k_{c}}^{i},z_{t,k_{c}}^{i},{\dot{z}}_{t,k_{c}}^{i}\right]}^{T}$, where $\left[x_{t,k_{c}}^{i},y_{t,k_{c}}^{i},z_{t,k_{c}}^{i}\right]{\;}^{T}$ represents the position, and $\left[{\dot{x}}_{t,k_{c}}^{i},{\dot{y}}_{t,k_{c}}^{i},{\dot{z}}_{t,k_{c}}^{i}\right]{\;}^{T}$ is the velocity at the frame capture instant $k_{c}$.

The first phase to model this problem involves obtaining the *t*-th target position measurements $z_{t,k_{c},3D}^{i}$ in 3D space for the targets at the *i*-th camera node from intersecting *LoSs* measured asynchronously in neighboring camera nodes. Subsequently, at each camera node, position and velocity estimation are initialized for each target. The local estimation algorithms are the Bayesian filters BAF and particle filtering (PF) without engaging in the fusion step though [15,17]. They compute the probability density function of the target *pdf* $p(x_{t,k_{c},3D}^{i,i}|z_{t,1:k_{c},3D}^{i})$ based on the known predicted posterior *pdf* $p({\hat{x}}_{t,k_{c},3D}^{i}|z_{t,1:k_{c}-1,3D}^{i})$ corresponding to the previous capture instant $k_{c}-1$ and propagated to capture instant $k_{c}$, the state transition *pdf* $p(x_{t,k_{c},3D}^{i,i}|{\hat{x}}_{t,k_{c}-1,3D}^{i})$, and the measurement likelihood *pdf* $p(z_{t,k_{c},3D}^{i}|x_{t,k_{c},3D}^{i,i})$. The second superscript in *i,i* refers to the local state estimate processed at the *i*-th camera not using information received from a neighboring camera.

We consider the frame processing delay incurred to obtain $z_{t,k_{c},3D}^{i}$ and the asynchrony of neighboring camera capture instants. To this end, we borrow from [15] the concept of a time window $K_{k}^{i}$ about the local camera capture instant $k_{c}$, wherein *LoS* vectors are received from neighboring cameras. We define $\tau_{k_{c}}^{i}$ as the frame processing delay to obtain the measurement $z_{t,k_{c},3D}^{i}$ for the *i*-th camera, and $\alpha^{ij}$ is the relative offset between the capture instants of neighboring cameras $C^{i}$ and $C^{j}$.

The problems arising with false positives and false negatives in target detection were considered previously [28] and are addressed here as well. Scene complexity (quantity and speed of the targets) [29], construction parameters of the cameras, and accuracy in their calibration [30,31,32] are factors that also hinder target detection. Thus, if the detection algorithm fails to confirm the presence of the same target within the limits of a minimum capture count up to a maximum count, then the target is discarded. A new identifier number (*Id*) assignment is created, and a new initial target centroid measurement in the image plane, expressed in pixels, is generated when detecting a novel target. We assume the detection algorithm does not incur in a frame processing delay [33] to obtain a target centroid measurement in the image plane, and the delay is solely from computing the position measurement $z_{t,k_{c},3D}^{i}$ in 3D space with the received *LoS* vectors transmitted by neighboring camera nodes.

As stated earlier, the cameras will fuse information from targets detected in their *FoVs* with information received from the targets of neighboring cameras belonging to $N^{j,i}$. Recall the *LoS* vector from a single pinhole camera model to a target does not convey depth information for tracking in 3D space [32]. Then, subject to network asynchrony, *j*-th cameras in the *i*-th camera neighborhood set $N^{j,i}$ transmit *LoS* vectors to distinct targets, each target being detected by at least two cameras with overlapping *FoVs* [30]. We use stereoscopy to measure depth along a *LoS* vector and determine 3D position as targets move freely. Stereoscopy must have views to the same target from at least two different directions. The *LoS* vector from the camera’s optical center to the target centroid in the image plane is represented in the global Cartesian coordinate system by means of camera calibration and pose [31] data. In a real scenario, the same target may have very distinct portions of its surface viewed from very different directions by cameras located far apart. In such a case, stereoscopy can produce a biased 3D position measurement located somewhere inside the target surface. A distance tolerance $d_{min}\leq \delta$ is established to detect *LoS* proximity and ascertain that the measured 3D position is consistent with the pair of *LoS*s involved.

We evaluate three approaches to information exchange:

- First: We propose to exchange among neighboring cameras *LoS* vector measurements ${\vec{LoS}}_{t,k_{c}+\tau_{k_{c}}^{i},i}^{w}$ to the *t*-th target from the *i*-th camera at instant $k_{c}+$ $\tau_{k_{c}}^{i}$ within the time window $K_{k}^{i}$ period, and the data are represented in the world coordinate system {W}. The measured $z_{t,k,2D}^{i}$ (referring to the target centroid in the image plane in pixel coordinates) is transformed into ${\vec{LoS}}_{t,k,i}^{w}$ with the camera construction parameters as described in Section 2.1. The fused 3D position measurement $z_{t,k_{c},3D}^{i}$ results from averaging intersecting pairs of vector constraints ${\vec{r}}_{t,k,i}^{w},\;{\vec{r}}_{t,k,j}^{w}$ derived from *LoS* vectors of neighboring *j*-th cameras pointing at the same *t*-th target according to a *LoS*-vector 3D proximity determination algorithm. Local target state estimation uses the fused 3D position measurement $z_{t,k_{c},3D}^{i}$ to update and predict the state estimate in the next frame capture instant $k_{c}+1$.
- Second: For comparison, exchange among cameras $C^{i}$ and $C^{j}$, where $j\;\in N^{j,i}$, the 3D fused measurement $z_{t,k_{c},3D}^{i}$ for each target, transmitted and instantaneously received at instants within the fusion window with configurable *p* size after the $K_{k}^{i}$ time window. This is the used in a sequential approach in SA-PF.
- Third: Again, for comparison, exchange among cameras $C^{i}$ and $C^{j}$, where $j\;\in N^{j,i},\;j\neq i$, information pairs the estimates for each target, transmitted and instantaneously received at instants within the fusion window with configurable *p* size after the $K_{k}^{i}$ time window. This is used in the batch approach in BAF.

The first approach extends the concept of stereoscopy to neighboring calibrated cameras with known poses and requires a 3D proximity metric among locally detected *LoS* vectors in the *i*-th camera and *LoS* information received from cameras belonging to $N^{j,i}$. Note that internal temporary storage of *LoS* vectors ${\vec{LoS}}_{t,k,i}^{w}$ at each instant within the time window $K_{k}^{i}$ period is required because the *LoS* vectors ${\vec{LoS}}_{t,k,i}^{w}$ at $C^{i}$ must be aligned temporally, through time stamps, with the vectors ${\vec{LoS}}_{t,k,j}^{w}$ received from $C^{j}$. In [34], the concept of “virtual synchronization” was used along a “dynamic” memory to store the captured data. Based on this concept, sending and receiving information from cameras are concurrent activities at instants k within the time window period.

The information vector $I_{t,k_{c}+\tau_{k_{c}}^{i},i}^{w}$ exchanged between cameras conveys the *LoS* vector to the *t*-th target at the time instant $k_{c}+\tau_{k_{c}}^{i}$, the time stamp referring to the $k_{c}+\tau_{k_{c}}^{i}$ instant and the optical center $O_{c,i}^{w}$ of camera $C^{i}$ expressed in world coordinates {W}: $I_{t,k_{c}+\tau_{k_{c}}^{i},i}^{w}=\{k_{c}+\tau_{k_{c}}^{i};O_{c,i}^{w};{\vec{LoS}}_{t,k_{c}+\tau_{k_{c}},i}^{w}\}$; the information vector stored for each of the k instants within the time window $K_{k}^{i}$ period is defined as $i_{t,k,i}^{w}=\{k;O_{c,i}^{w};{\vec{LoS}}_{t,k,i}^{w}\}$. We adopt capital ***I*** for the exchanged information vector and small ***i*** for the stored information vector.

### 2.1. Obtaining $z_{t,k_{c},3D}^{i}$

To obtain measurements in 3D space, the following steps are required, from capturing the target centroid in the image plane $z_{t,k,2D}^{i}$ in pixels to measuring the centroid $z_{t,k_{c},3D}^{i}$ in 3D space in meters.

#### 2.1.1. Target Centroid Detection in the Image Plane in Pixels and Identification Number (*Id*) Assignment

**Figure 1:**
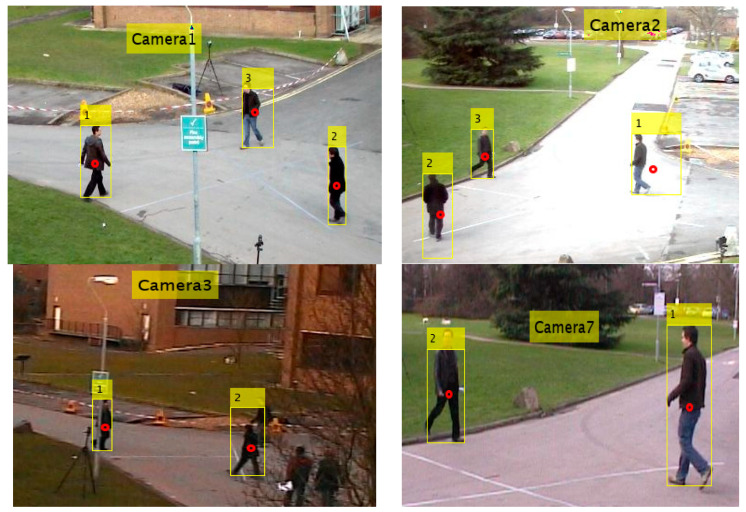
Targets detected within the *FoVs* of Cameras 1, 2, 3, and 7 and the assigned corresponding assigned identification numbers [27]. Red dots indicate centroids of each target.

For each target detected by the *i*-th camera, the target centroid in the image plane is represented in the image by a red dot, as shown in Figure 1. All targets are assigned an identification number (*Id*).

Concerning the detection algorithm, accurate data association for maintaining the assignment of an identifier to a target is a challenging problem. The “global nearest neighbor” algorithm [35], which in turn implements the Munkres algorithm [36], was used at each camera for this purpose. However, as shown in Figure 1, an *Id* assigned to a target by one of the cameras will not necessarily match the *Id* assigned to the same target by neighboring cameras. For example, in Figure 1, for the same target observed by Cameras 1 and 2, Camera 1 assigned *Id* = 1 while Camera 2 assigned *Id* = 3. This issue is addressed in Section 2.3.

Centroid pixels in Figure 1 should be represented in the corresponding camera {C} coordinate system, and the cameras’ calibration will correct the distortions caused by camera construction parameters.

#### 2.1.2. Camera Calibration

**Figure 2:**
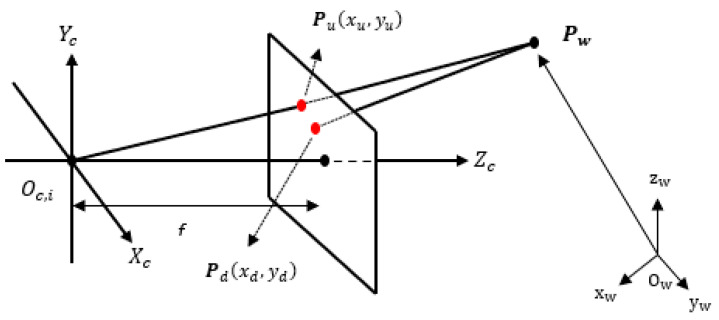
Pinhole camera model $C^{i}$: {C} represents the camera coordinate system; {W} represents the world coordinate system; $O_{c,i}$ represents the optical center of the *i*-th camera expressed in the camera coordinate system; $O_{w}$ represents the origin of the world coordinate system; **f** is the focal length; $P_{d}$ is the distorted point given in pixels; $\;P_{u}$ represents the corrected point given in pixels; $P_{w}$ is a point in the 3D space represented in world coordinates {W}; $Z_{c}=f$ is the camera image plane.

All cameras follow the pinhole CCD model [32]. Using intrinsic or construction parameters of a camera, it is possible to correct its distortions and calibrate it so that the distorted pixel of the detected centroid of the *t*-th target, represented by point $P_{d}$ in Figure 2, corresponds to the corrected position in the image plane represented by point $P_{u}$. The transformation $(x_{d},y_{d})\to \left(x_{u},y_{u}\right)$ follows [37].

After correcting point $P_{d}$ to point $\;P_{u}$ in the camera coordinate system {C}, we represent two main points in the world coordinate system {W} for each camera: the optical center point $O_{c,i}$ and point $\;P_{u}$ that give rise to the *LoS* vector from the *i*-th camera to the *t*-th target.

#### 2.1.3. The Optical Center $O_{c,i}$ and Point $P_{u}$ Represented in the World Coordinate System {W}: A Preamble for Obtaining the *LoS* Vector

Given that the extrinsic parameters of camera $C^{i}$ represent its rotation $R^{i}$ and translation ${\vec{t}}^{i}$**,** the representation of the optical center $O_{c,i}$ defined as vector ${\tilde{C}}^{i}=O_{c,i}$ [32], represented in camera coordinate system {C}, in world coordinate system {W} is expressed as follows:

(1) {C}→{W}→Oc,iw=t→cwi=−Rcwi′C˜i

Point $P_{u}$ is located in image plane, as shown in Figure 2. We must first represent the pixels of $P_{u}$ in coordinates of the camera system {C}:

(2) $$\left\{pixels\right\}\to \left\{C\right\}\to \left(x_{u}=f\frac{x_{c}}{z_{c}},y_{u}=f\frac{y_{c}}{z_{c}},z_{c}=f\right)\to \left(x_{u}=x_{c},\;y_{u}=y_{c},f\right)$$

Denoting the *LoS* vector $X_{t,c}^{i}=\left[x_{c}=x_{u},y_{c}=y_{u},f\right]$ as the coordinates obtained from point $P_{u}$ for the *t*-th target, now represented in coordinates of the camera system {C}, we can represent it in a vector base with origin at the camera optical center $O_{c,i}$ and parallel to the world coordinate system {W} as follows:

(3) {C}→{W}→LoS→t,k,iw=Rcwi′Xt,ci

The rotation matrix Rcwi is expressed as follows:

(4) Rcwi(θz,θy,θx)=[cosθycosθzcosθzsinθxsinθy−cosθxsinθysinθxsinθz+cosθxcosθzsinθycosθysinθzsinθxsinθysinθz+cosθxcosθzcosθxsinθysinθz−cosθzsinθx−sinθycosθysinθxcosθxcosθy]

This is the matrix that performs the rotation from system {W} to system {C} for each camera; its rotation order in camera axes is zyx and angles $\theta_{z},\theta_{y},\theta_{x}$ rotate from {C} to {W} [31], then it is necessary to apply the transpose operation (′) in Equations (1) and (3).

#### 2.1.4. *LoS* Vector ${\vec{LoS}}_{t,k,i}^{w}$ and Parametric Equation of the Line

**Figure 3:**
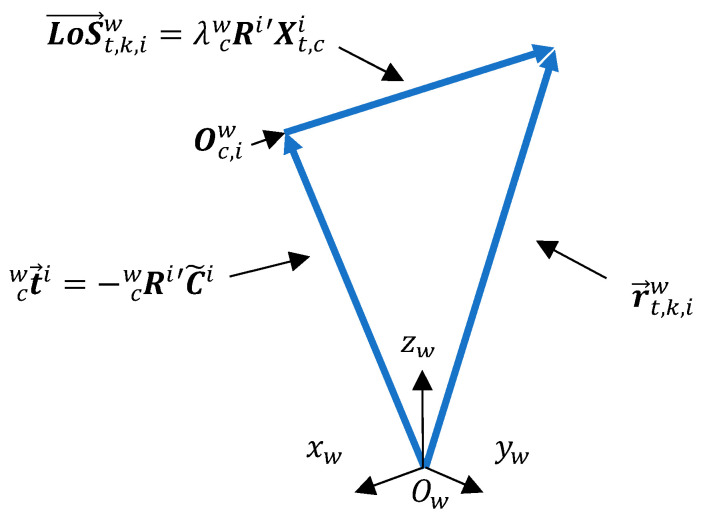
Camera optical center and *LoS* vector representation in the world coordinate system {W}. The constraint vector ${\vec{r}}_{t,k,i}^{w}$ results from summing the translation vectors t→cwi and the extended *LoS* vector $\lambda {\vec{LoS}}_{t,k,i}^{w},\;\lambda \in \mathbb{R},\;\lambda >1$.

Note in Figure 3 that the *LoS* vector ${\vec{LoS}}_{t,k,i}^{w}$ is simply the point $X_{t,c}^{i}$ multiplied by the transposed rotation matrix of the *i*-th camera according to Equation (3).

It should be noted that t→cwi is a constant vector because it refers to the fixed position of the optical center of the *i*-th camera. The *LoS* vector, in turn, depends on the position of the target, referring to point $X_{t,c}^{i}$, in the image plane expressed in camera coordinates system {C}. Writing the constraint vector ${\vec{r}}_{t,k,i}^{w}$ in the form of a parametric equation [38] yields the following expression:

(5) r→t,k,iw(λ)=t→cwi+λLoS→t,k,iw,  λ∈ℝ, λ>1

**Figure 4:**
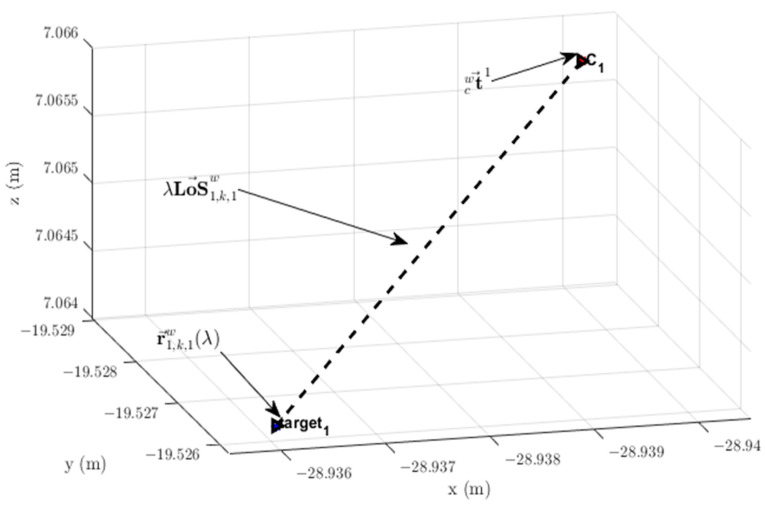
*LoS* vector ${\vec{LoS}}_{1,k,1}^{w}$ and respective vector constraint ${\vec{r}}_{1,k,1}^{w}\left(\lambda \right)$ from Camera 1 to the detected target with Id=1. C1=t→cw1: location of the optical center of Camera 1. Target 1: position of Target 1 in the image plane giving rise to *LoS* vector ${\vec{LoS}}_{t,k,i}^{w}$.

Thus, the vector constraint ${\vec{r}}_{t,k,i}^{w}\left(\lambda \right)$ changes when extending the *LoS* vector ${\vec{LoS}}_{t,k,i}^{w}$ with parameter λ. Figure 4 shows the *LoS* vector ${\vec{LoS}}_{t,k,i}^{w}$ and points related to vectors constraint ${\vec{r}}_{t,k,i}^{w}\left(\lambda \right)$ and optical center position t→cwi to Target 1 on Camera 1. The last two vectors have their origins coinciding with the origin {0;0;0} of the world coordinate system {W}.

#### 2.1.5. Proximity Metric between Vector Constraints Originating from *LoS* to a Target

Assume camera $C^{i}$ receives the information vector $I_{t,k_{c}+\tau_{k_{c}}^{j},j}^{w}$ from one of the transmitting cameras $C^{j}$. Suppose also that the observed scene contains only one target, and two synchronized cameras—$C^{i}$ and $C^{j}$ with $\alpha^{ij}=0$—observe it with overlapping *FoVs* at the same time of capture instant in both cameras; moreover, there is no image processing delay, i.e., $\tau_{k_{c}}^{i}=\tau_{k_{c}}^{j}=0$. In these circumstances, we adopt for simplicity here subscript *k* to mean the capture instant $k_{c}$. The vector constraints ${\vec{r}}_{t,k,i}^{w}$ and ${\vec{r}}_{t,k,j}^{w}$, respectively, are computed from the information vectors of cameras $C^{i}$ and $C^{j}$, respectively, and can be written as follows:

(6) r→t,k,iw(λ)=t→cwi+λLoS→t,k,iw, λ ∈ℝ, λ>1r→t,k,jw(β)=t→cwj+βLoS→t,k,jw, β∈ℝ, β>1

Then, target triangulation must find λ*,β*=minλ,βd(r→t,k,iw(λ),r→t,k,jw(β))=minλ,β d(t→cwi+λLoS→t,k,iw ,t→cwj+βLoS→t,k,jw) that minimize the distance between vector constraints ${\vec{r}}_{t,k,i}^{w}\left(\lambda \right)$ and ${\vec{r}}_{t,k,j}^{w}\left(\beta \right)$. The concept of distance between reversed straight lines is applied [38,39], and the solution is obtained by calculating the partial derivative of the distance with respect to λ and β.

(7) $$\{\begin{array}{c} \frac{\partial d\left({\vec{r}}_{t,k,i}^{w}\left(\lambda \right),{\vec{r}}_{t,k,j}^{w}\left(\beta \right)\right)}{\partial \lambda }=0\\ \frac{\partial d\left({\vec{r}}_{t,k,i}^{w}\left(\lambda \right),{\vec{r}}_{t,k,j}^{w}\left(\beta \right)\right)}{\partial \beta }=0 \end{array}.$$

Solving (7) produces the optimized nonlinear parameters expressed as follows:


(8) {λ*= (LoS→t,k,iw·LoS→t,k,jw)[(t→cwi−t→cwj)·LoS→t,k,jw]−||LoS→t,k,jw||2[(t→cwi−t→cwj)·LoS→t,k,iw]||LoS→t,k,iw||2||LoS→t,k,jw||2−(LoS→t,k,iw·LoS→t,k,jw)2β*=(t→cwi−t→cwj)·LoS→t,k,jw+λ*(LoS→t,k,iw·LoS→t,k,jw)||LoS→t,k,jw||2

The operator · represents the inner product between the vectors, and ||.|| represents the vector 2-norm operator. Appendix A shows how to find the optimized parameters $\;\lambda^{*}\;\mathrm{and}\;\beta^{*}$.

**Figure 5:**
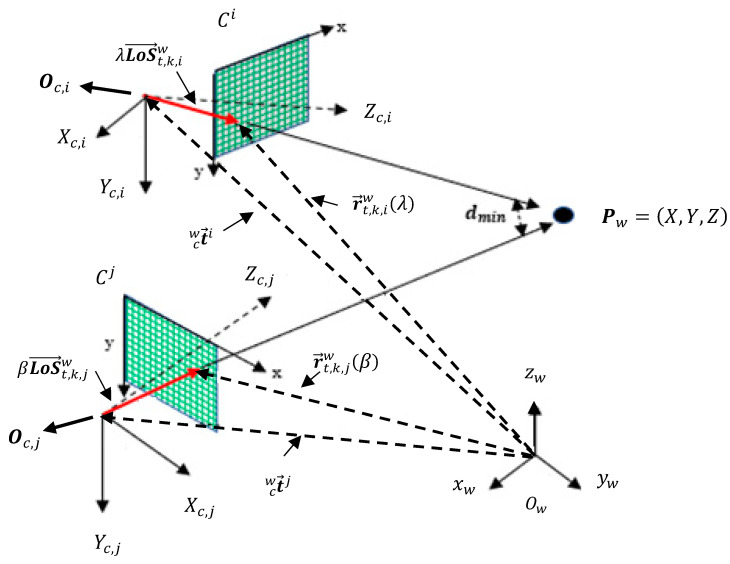
Determining the distance between vector constraints ${\vec{r}}_{t,k,i}^{w}\left(\lambda \right)$ and ${\vec{r}}_{t,k,j}^{w}\left(\beta \right)$. The tolerance $d_{min}\leq \delta$ indicates whether *LoS* vectors ${\vec{LoS}}_{t,k,i}^{w}$ and ${\vec{LoS}}_{t,k,j}^{w}$ point at the same target.

Once obtained, these parameters are substituted in (6), and the minimum distance occurs where the 3D position is in ${\vec{r}}_{t,k,i}^{w}\left(\lambda^{*}\right)$. A tolerance $d_{min}\leq \delta =0.3$ m was chosen to indicate whether the pair of *LoS* vectors point at the same target. Figure 5 shows *LoS* vectors ${\vec{LoS}}_{t,k,i}^{w}$ and ${\vec{LoS}}_{t,k,j}^{w}$ pointing at the same target.

The above description concerns information vectors exchanged synchronously. Next, we analyze asynchronous information exchange considering the processing time required for obtaining position measurements in 3D space.

#### 2.1.6. Asynchronous Exchange of Information Vectors

**Figure 6:**
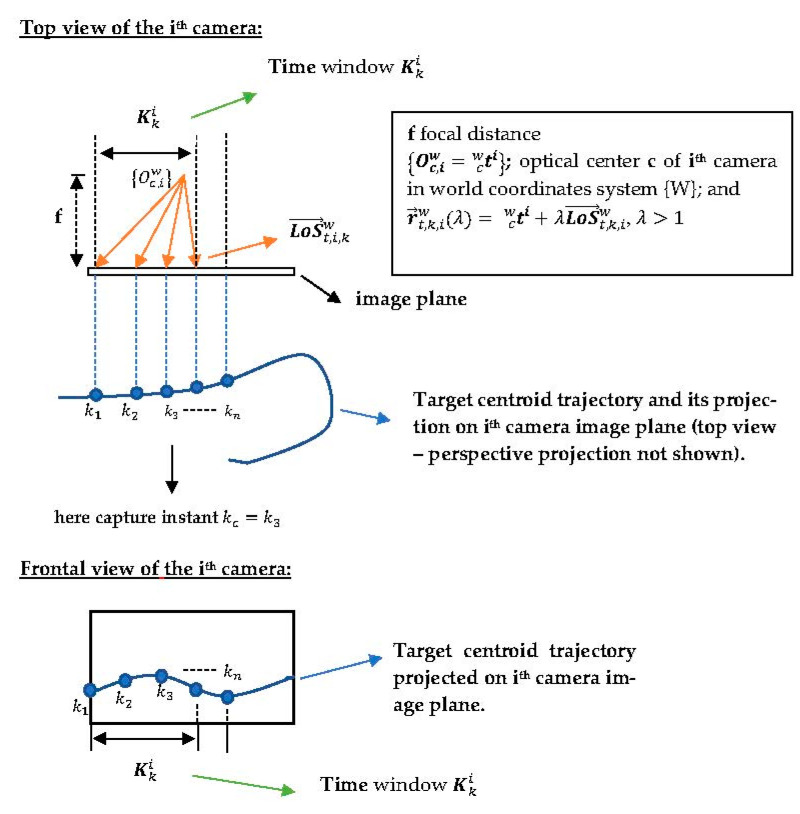
Top and front views of a detected target centroid trajectory within a time window $K_{k}^{i}$.

Figure 6 shows the top and front views of the *t*-th target centroid trajectory during time window $K_{k}^{i}$ in one of the cameras, assuming $k_{c}=k_{3}\;$ to be the frame capture instant. Both the optical center $O_{c,i}^{w}$ and the *LoS* vector ${\vec{LoS}}_{t,k,i}^{w}$ that define the vector constraint ${\vec{r}}_{t,k,i}^{w}\left(\lambda \right)$ are observed in Figure 6. The frame processing delay of the detection algorithm is assumed negligible relative to acquiring the 3D position measurements of targets’ centroids instants k within the time window $K_{k}^{i}$.

**Figure 7:**
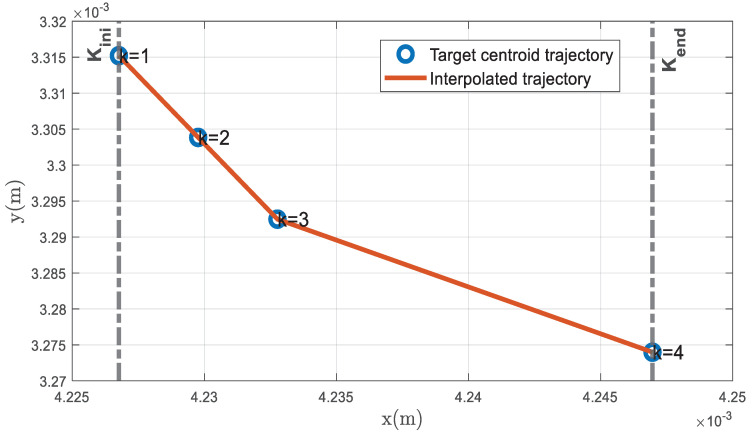
*LoS* vectors from Camera 1 to Target 1 ${\vec{LoS}}_{1,k,1}^{w},\;k=1,\ldots ,4$ in the image plane in {W} coordinates during the time window $K_{k}^{1}$ and the interpolated trajectory. The data will be stored in the information vectors $i_{1,k,1}^{w}=\{k;O_{c,1}^{w};{\vec{LoS}}_{1,k,1}^{w}\}$ for each *k*-th instant within the time window $K_{k}^{1}$.

An example of a target centroid trajectory in the image plane expressed in world coordinates {W} is depicted in Figure 7.

**Figure 8:**
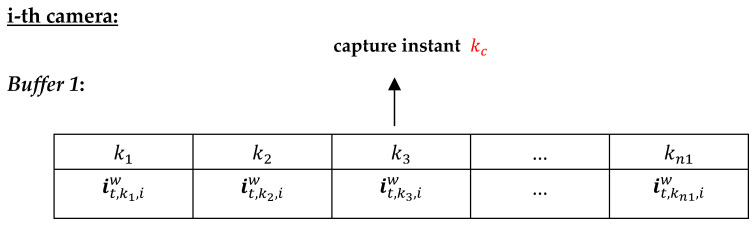
Information vector storage buffer $i_{t,k,i}^{w}$ of the *t*-th target in the *i*-th camera, $k=k_{1},\ldots ,k_{n1}$.

Following an idea reported in [34], the information vectors $i_{t,k,i}^{w}$ are stored for each *k*-th instant within the time window $K_{k}^{i}$, as can be observed in *Buffer 1* of the *i*-th camera in Figure 8. They are subsequently replaced by information vectors of the next time window $K_{k+1}^{i}$. Note that to each detected *t*-th target a buffer is allocated to store its information vectors.

**Figure 9:**
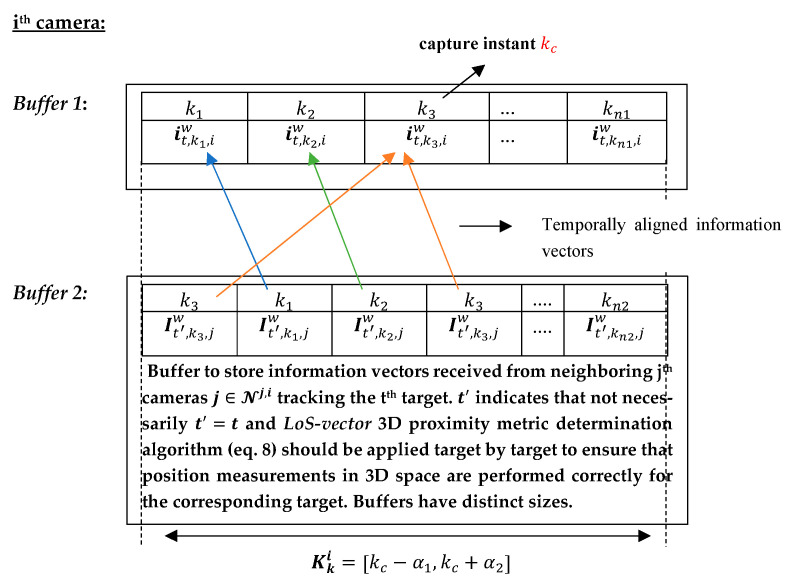
*Buffer 1*: locally obtained information vectors stored in the *i*-th camera. *Buffer 2*: information vectors received from neighboring cameras at different time instants within the time window period. The information vectors are temporally aligned for later 3D proximity metric computation. Reception of two or more information vectors may occur at the same time instant within the time window $K_{k}^{i}$, as shown at time $k_{3}$. Colored arrows indicate temporal alignment of local information vectors with received information vectors.

Information vectors are sent and received by the *i*-th camera within the time window about the capture instant $k_{c}$, expressed as $K_{k}^{i}=\left[k_{c}-\alpha_{1},k_{c}+\alpha_{2}\right]$, $\left(\alpha_{1}>0\;\mathrm{and}\;\alpha_{2}>\tau_{k_{c}}^{i}\right)$. The information vectors $i_{t,k,i}^{w}$ of the *t*-th target in the *i*-th camera (*Buffer 1*) are aligned according to their time stamps with respect to time stamps in the information vectors of various targets received from the *j*-th neighboring cameras (*Buffer 2*). After the temporal alignment, $C^{i}$ will contain local information vectors (*Buffer 1*) and received information vectors from the *j*-th neighboring cameras (*Buffer 2*), as depicted in Figure 9. 

After temporal synchronization of the information vectors, the proximity metric algorithm is initialized to determine the distance between constraint pairs ${\vec{r}}_{t,k,i}^{w}$ and ${\vec{r}}_{t,k,j}^{w}$. The 3D position measurement in 3D space $z_{t,k,3D}^{i}$ results from local constraints ${\vec{r}}_{t,k,i}^{w}$ and constraints ${\vec{r}}_{t,k,j}^{w}$ received from neighboring cameras within each time window $K_{k}^{i}$ that are found to point to the same *t*-th target at the same instant *k* when distance between the constraint pair is less than tolerance δ. A polynomial interpolates the 3D position measurements of the same target $z_{t,k,3D}^{i}$, propagating these 3D measurements backward or forward in time within the time window $K_{k}^{i}$ until alignment with the *i*-th camera captures instant $k_{c}$, and fuses these 3D measurements by averaging them, thus producing the fused 3D position measurement $z_{t,k_{c},3D}^{i}$. An internal variable $N_{c}$ defined for each *t*-th target counts the number of cameras tracking the same target for the purpose of averaging. Then follows local estimation via update with measurement $z_{t,k_{c},3D}^{i}$ and time propagation to the next capture instant, either with BAF or SA-PF, without engaging in any further fusion phase and hence reducing the communication load.

**Figure 10:**
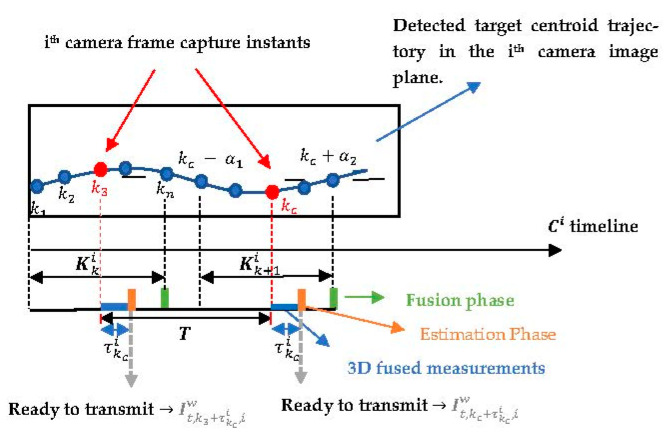
Front view of the *i*-th camera with a detected target centroid trajectory. $\tau_{k_{c}}^{i}$ denotes the image processing delay incurred measuring $z_{t,k_{c},3D}^{i}$ and the local estimation phase. $T=\alpha_{1}+\alpha_{2}$ is the inter-frame interval. $K_{k}^{i}=\left[k_{c}-\alpha_{1},k_{c}+\alpha_{2}\right]$: time window. Transmission of information vectors $I_{t,k_{c}+\tau_{k_{c}}^{i},i}^{w}$ occurs at $k_{c}+\tau_{k_{c}}^{i}$ whereas the fusion phase occurs at the end of the time window, i.e., at $k_{c}+\alpha_{2}$. Instants *k* are integer multiples of the frame capture period *T*.

For comparison, the fusion window takes place at the end of each time window $K_{k}^{i}$ at time $\left(k_{c}+\alpha_{2}\right)$, either using information pairs’ estimates in BAF or fused 3D position measurements in SA-PF received asynchronously from neighboring cameras. As described in [15], each camera node knows the maximum relative offset $\alpha^{max}=\underset{i,j}{\max}\left\{\alpha^{ij}\right\}$ and the maximum and minimum processing delays $\tau^{max}=\underset{i,k_{c}}{\max}\left\{\tau_{k_{c}}^{i}\right\}$ e $\tau^{min}=\underset{i,k_{c}}{\min}\left\{\tau_{k_{c}}^{i}\right\}$. To avoid the next capture instant $k_{c}$ coinciding with the fusion phase of the previous time window, a separation $T=\alpha_{1}+\alpha_{2}$ between consecutive capture instants must exist where $\alpha_{1}=\alpha^{max}-\tau^{min}$ and $\alpha_{2}=\alpha^{max}+\tau^{max}$ [15]. Figure 10 shows the timeline that describes how the actions elapse within time window $K_{k}^{i}$. The algorithm that obtains the 3D position measurements takes into account the information vectors $I_{t,k_{c}+\tau_{k_{c}}^{i},j}^{w}$ exchanged at the time window within the period $\left[k_{c}-\alpha_{1},k_{c}+\tau_{k_{c}}^{i}\right]$. The estimation phase takes place at $k_{c}+\tau_{k_{c}}^{i}$ using the fused 3D position measurement $z_{t,k_{c},3D}^{i}$. 

An important point addressed next is how to model the uncertainties inherent to the whole process described above and thus find the fused 3D position measurement $z_{t,k_{c},3D}^{i}$. 

#### 2.1.7. Obtaining the 3D Position Measurement at Capture Instant $k_{c}$

**Figure 11:**
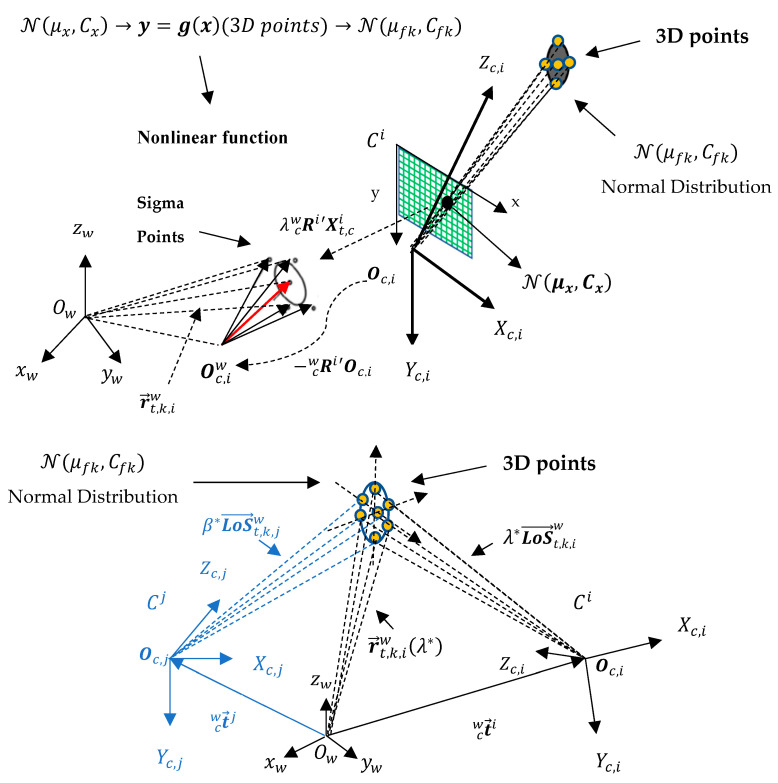
Application of the UT transformation to approximate the 3D position measurement error statistics arising from the uncertainty in *LoS* vector ${\vec{LoS}}_{t,k,i}^{w}$ in the image plane. This uncertainty in the *LoS* vector affects the output error statistics of the proximity metric algorithm that determines the distance between constraint vector pairs ${\vec{r}}_{t,k,i}^{w}$ and ${\vec{r}}_{t,k,j}^{w}$ (the latter not shown for the sake of clarity) and consequently impacts the 3D position measurement. The resulting seven points in 3D space are approximated with a normal distribution $N\left(\mu_{fk,}C_{fk}\right)$. Subscript *f* here means “final” in the UT 3D position mean and covariance determined from averaging with respect to the neighboring cameras tracking the same target centroid at the same time instant for each instant *k* within a temporal window. 3D motion of the imaged targets is not constrained to a ground reference plane. 3D position is in ${\vec{r}}_{t,k,i}^{w}\left(\lambda^{*}\right)$ when the distance tolerance between ${\vec{r}}_{t,k,i}^{w}\left(\lambda^{*}\right)$ and ${\vec{r}}_{t,k,j}^{w}\left(\beta^{*}\right)$ is $d_{min}\leq \delta$.

In the initialization of the proximity metric algorithm to determine the distance between constraint pairs ${\vec{r}}_{t,k,i}^{w}$ and ${\vec{r}}_{t,k,j}^{w}$, we model the *LoS* vector ${\vec{LoS}}_{t,k,i}^{w}$ with the “Unscented Transform” (UT) [19,40] to approximate the statistics of the random *LoS* variables transformed by the nonlinear function that determines depth from stereo. The uncertainty in the image plane is a three-dimensional Gaussian distribution $N\left(\mu_{x},C_{x}\right)$ that also accounts for camera calibration error in the focal length f, where $\mu_{x}$ is the mean, and $C_{x}$ is the covariance. This Gaussian distribution defines seven sigma points [40]. Figure 11 shows the 3D sigma points along the three axes of the *i*-th camera due to uncertainty in the detected centroid location in the image plane that gives rise to the *LoS* vector LoS→t,k,iw=Rcwi′Xt,ci represented in the world coordinate system {W}.

**Figure 12:**
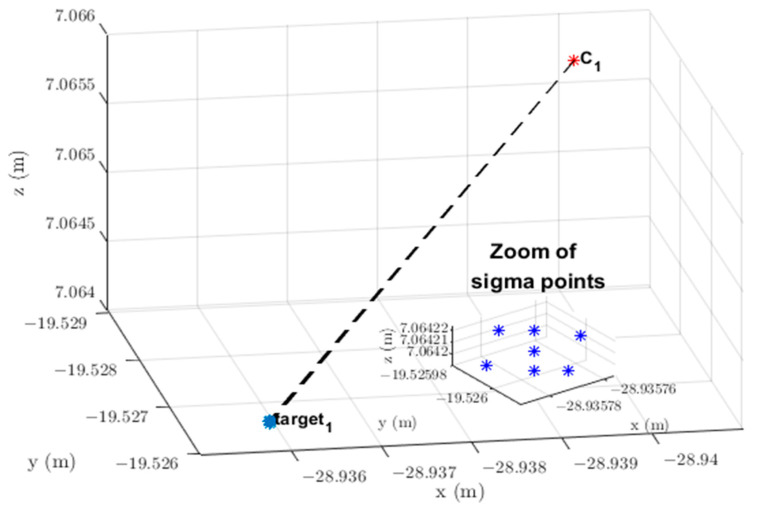
7 Sigma points found after applying UT to noisy *LoS* vectors in the image plane (optical axis included) undergoing the proximity metric algorithm to determine the distance between vector constraint pairs and the corresponding set of points in 3D space with distribution $N\left(\mu_{fk},C_{fk}\right)$.

Given that the dimension of $X_{t,c}^{i}$ is L=3, then 2L+1=7 sigma points in three-dimensions are generated in the image plane and along the optical axis, as shown in Figure 12. Consequently, seven constraints ${\vec{r}}_{t,k,i}^{w}$ are found for the same *t*-th target. The sigma points in the image plane are generated for each target from information that camera $C^{i}$ either detects locally or receives information about from a neighboring camera $C^{j}$. The mean $\mu_{x}$ of the Gaussian distribution is the *LoS* vector LoS→t,k,iw=Rcwi′Xt,ci shown in Figure 11, represented by a red arrow.

**Figure 13:**
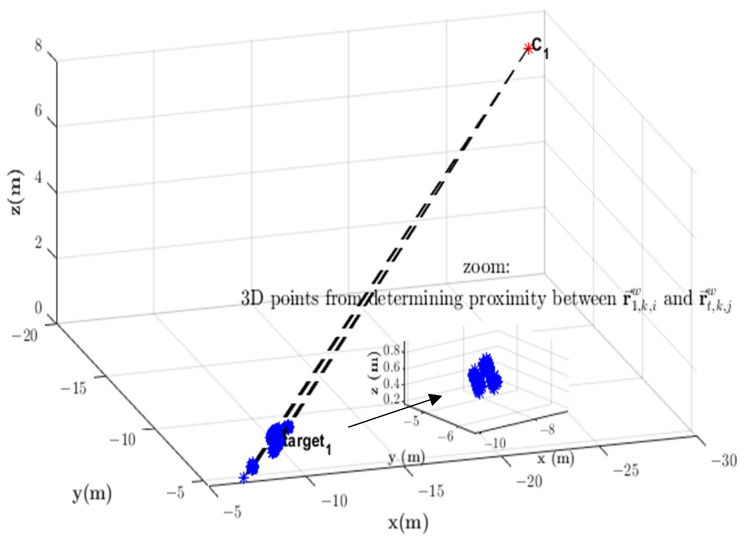
Set of 3D points resulting from proximity determination between constraints ${\vec{r}}_{1,k,i}^{w}$ and ${\vec{r}}_{t,k,i}^{w},\;j\;=\;2,3,4,5$ for target *Id* = 1 in Camera 1. Information vectors received from neighboring cameras can include *LoS* vectors to different targets or to the same target but with a different *Id* from the one issued in camera 1.

The proximity metric determines the distance (a nonlinear function) between each candidate constraint pair, i.e., ${\vec{r}}_{t,k,i}^{w}$ and ${\vec{r}}_{t,k,j}^{w}$ (amounting to a total of 49 candidate pairs, but only the 7 points with minimum distance are considered for each constraint pair). Any point in 3D space along constraint ${\vec{r}}_{t_{a},k,i}^{w}$ whose distance to another candidate constraint ${\vec{r}}_{t_{b},k,j}^{w}$ falls within the proximity tolerance $d_{min}\leq \delta$ indicates that targets $t_{a}$ seen by $C^{i}$ and $t_{b}$ seen by $C^{j}$ are the same target, and a set of points in 3D space are thus obtained. Figure 13 shows the result of proximity determination between a constraint to a target detected locally by Camera 1 and each of the constraints to targets received in information vectors from neighboring cameras. In this case, Cameras 2, 3, 4, and 5 send constraints ${\vec{r}}_{t,k,j}^{w}$ to Camera 1 within time window $K_{k}^{1}$ and some of them are relative to the same Target 1. 

Each set of 3D points with corresponding UT mean and covariance ($\mu_{fk},C_{fk})$ after averaging refers to a time instant within the time window $K_{k}^{i}$. Notice that more than one such set may relate to the same instant *k* when the UT processes simultaneous constraints that originate in information vectors transmitted by *n* neighboring cameras tracking the same target at instant *k*. The representative point in 3D space and respective covariance at instant *k*, $\mu_{fk}$, and $C_{fk}$ are the average over the *n* UT means and covariances $\mu_{jk},C_{jk},\;j\in N^{j,i}$ of simultaneous sets of points in 3D space at instant *k*. 

The final 3D mean, $\mu_{fk}$, averages over the *n* simultaneous UT means $\mu_{1k},\ldots ,\mu_{nk}$ in 3D space at instant *k* as follows:

(9) $$\mu_{fk}=\frac{\mu_{1k}+\mu_{2k}+\ldots +\mu_{nk}}{n}.$$

**Figure 14:**
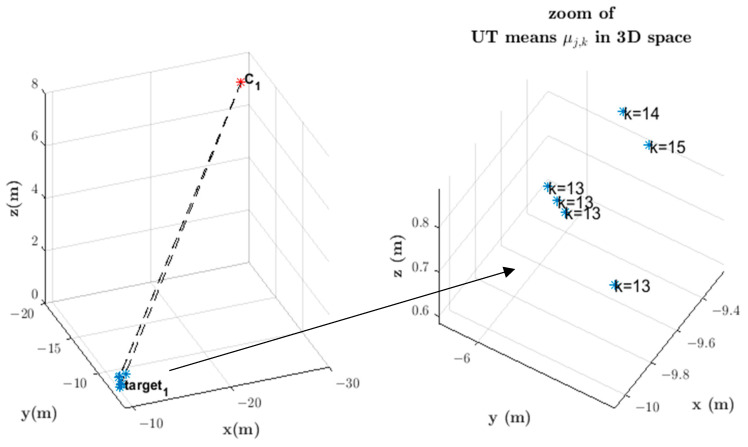
An instance of distinct UT means $\mu_{jk},\;j=1,\ldots ,n$ in 3D space at instant k for the sets of 3D points shown in Figure 13.

**Figure 15:**
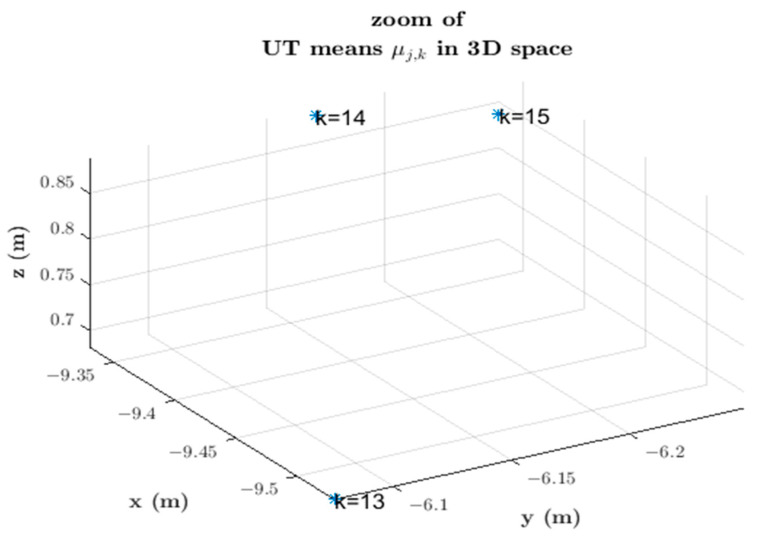
Final 3D means $\mu_{fk}$ at instants *k* = 13, *k* = 14, and *k* = 15 after averaging their respective simultaneous UT means $\mu_{jk}$ seen in Figure 14.

Likewise, $C_{fk}$ averages over simultaneous UT covariances $C_{1k},\ldots ,C_{nk}$. Figure 14 depicts this situation, where five UT means are obtained at instant k=13, one at instant k=14, and one at instant k=15. The result is the average of the UT means at each k time instant, as depicted in Figure 15. The corresponding UT covariance average is not shown in Figure 15.

Thus, 3D points are measured at each instant k within period $\left[k_{c}-\alpha_{1},k_{c}+\tau_{k_{c}}^{i}\right]$ of the time window $K_{k}^{i}$. Two conditions must be met for these measurements to be generated: (1) at least one information vector $I_{t,k_{c}+\tau_{k_{c}}^{j},j}^{w}$ is received from a neighboring camera at any instant k within the time window period $\left[k_{c}-\alpha_{1},k_{c}+\tau_{k_{c}}^{i}\right]$, and (2) this information vector relates to the same target observed at the *i*-th camera. These two conditions are not always met; in that case, the 3D position of the target centroid is not measured at all instants within the aforementioned period. At least two 3D position measurements at different instants within the time window period $\left[k_{c}-\alpha_{1},k_{c}+\tau_{k_{c}}^{i}\right]$ are necessary to derive a linear polynomial and interpolate from the target 3D position measurements forward or backward in time until the capture instant $k_{c}$ to compute the fused 3D position measurement $z_{t,k_{c},3D}^{i}$. This interpolation process occurs at the instant $\;k_{c}+\tau_{k_{c}}^{i}$, and $z_{t,k_{c},3D}^{i}\;$ is the average of the 3D UT means measured within the period $\left[k_{c}-\alpha_{1},k_{c}+\tau_{k_{c}}^{i}\right]$ and interpolated in time until the capture instant $k_{c}$. The corresponding fused covariance $C_{t,k_{c},3D}^{i}$ is the average of UT covariances interpolated in time until the capture instant. 

In essence, our approach provides the fused 3D position measurement $z_{t,k_{c},3D}^{i}$ and respective covariance $C_{t,k_{c},3D}^{i}$ from the following steps: 

(1) The 3D position measurements $z_{t,k,3D}^{i}$ and covariances $C_{t,k,3D}^{i}$ are output by the *i*-th camera local proximity algorithm at each *k* instant within period $\left[k_{c}-\alpha_{1},k_{c}+\tau_{k_{c}}^{i}\right]$ if the two conditions above are met; 

(2) Then, a linear polynomial interpolation propagates $z_{t,k,3D}^{i}$ and $C_{t,k,3D}^{i}$ backward or forward in time until alignment with the *i*-th camera local capture instant $k_{c}$;

(3) Finally, the fused 3D position measurements $z_{t,k_{c},3D}^{i}$ and covariances $C_{t,k_{c},3D}^{i}$ result from averaging the interpolated measurements and covariances at capture instant $k_{c}$. 

A remark is that when just one 3D position measurement is generated at an instant that differs from $k_{c}$, the measurement is discarded because there is no other point to derive a linear interpolating polynomial, and the next time window is awaited. By contrast, when just a single 3D position measurement occurs at frame capture instant $k_{c}$, this measurement is accepted as $z_{t,k_{c},3D}^{i}$ with the corresponding covariance $C_{t,k_{c},3D}^{i}$. 

The fused 3D position measurement $z_{t,k_{c},3D}^{i}$ and covariance $C_{t,k_{c},3D}^{i}$ at instant $k_{c}$ are the required information for our decentralized approach with either the local estimation phase at instant $k_{c}+\tau_{k_{c}}^{i}$ or further fusion-based estimation with BAF or SA-PF at instant $k_{c}+\alpha_{2}$.

### 2.2. Dynamic Target Model: Linear Gaussian Model

The discrete-time dynamic model for each target [41] at each camera, is given by

(10) $$x_{t,k_{c}+\Delta k_{c},3D}^{i}=F\left(k_{c},k_{c}+\Delta k_{c}\right)x_{t,k_{c},3D}^{i}+w\left(k_{c},k_{c}+\Delta k_{c}\right)$$

where $F\left(k_{c},k_{c}+\Delta k_{c}\right)$ is the state transition matrix from instant $k_{c}$ to $k_{c}+\Delta k_{c}$, defined as

(11) $$F\left(k_{c},k_{c}+\Delta k_{c}\right)=\left[\begin{array}{cccccc} 1 & \Delta k_{c} & 0 & 0 & 0 & 0\\ 0 & 1 & 0 & 0 & 0 & 0\\ 0 & 0 & 1 & \Delta k_{c} & 0 & 0\\ 0 & 0 & 0 & 1 & 0 & 0\\ 0 & 0 & 0 & 0 & 1 & \Delta k_{c}\\ 0 & 0 & 0 & 0 & 0 & 1 \end{array}\right]$$

and $w\left(k_{c},k_{c}+\Delta k_{c}\right)$ is the cumulative noise between time instants $k_{c}$ and $k_{c}+\Delta k_{c}$, which is considered to be Gaussian with zero mean and covariance $Q\left(k_{c},\;k_{c}+\Delta k_{c}\right)$ expressed as follows:

(12) $$Q\left(k_{c},\;k_{c}+\Delta k_{c}\right)=\sigma_{u}^{2}\left[\begin{array}{cccccc} \frac{\Delta k_{c}{}^{3}}{3} & \frac{\Delta k_{c}{}^{2}}{2} & 0 & 0 & 0 & 0\\ \frac{\Delta k_{c}{}^{2}}{2} & \Delta k_{c} & 0 & 0 & 0 & 0\\ 0 & 0 & \frac{\Delta k_{c}{}^{3}}{3} & \frac{\Delta k_{c}{}^{2}}{2} & 0 & 0\\ 0 & 0 & \frac{\Delta k_{c}{}^{2}}{2} & \Delta k_{c} & 0 & 0\\ 0 & 0 & 0 & 0 & \frac{\Delta k_{c}{}^{3}}{3} & \frac{\Delta k_{c}{}^{2}}{2}\\ 0 & 0 & 0 & 0 & \frac{\Delta k_{c}{}^{2}}{2} & \Delta k_{c} \end{array}\right],$$

where $\sigma_{u}^{2}$ is the variance of process noise in the nearly-constant-velocity model [41]. The measurement model for the *i*-th camera is given by $z_{t,k_{c},3D}^{i}=Hx_{t,k_{c},3D}^{i}+\nu^{i}$, where $\nu^{i}$ is the measurement noise vector with dimensions 3 × 1, which is assumed to be Gaussian with zero mean and covariance matrix $R^{i}$. The latter is the fused covariance matrix $C_{t,k_{c},3D}^{i}$ resulting from interpolation into alignment with frame capture instant $k_{c}$ and averaging as described in Section 3.1, and H is given by

(13) $$H=\left[\begin{array}{cccccc} 1 & 0 & 0 & 0 & 0 & 0\\ 0 & 0 & 1 & 0 & 0 & 0\\ 0 & 0 & 0 & 0 & 1 & 0 \end{array}\right]$$

### 2.3. Target Information Fusion within the Fusion Window [15]

Our proposal is the use of fused 3D position measurements derived from stereoscopy in combination with local state estimation to track actual moving subjects undergoing free 3D motion within the fields of view of asynchronous, calibrated cameras with known poses and located far apart. In certain circumstances, very distinct parts of a target surface can be viewed from very different viewing directions; thus, the 3D centroid position of such a target lies at some point inside and not on the viewed surfaces. Local estimation uses either the Kalman filter in information form or particle filtering. Then we test additional fusion with either BAF or SA-PF for comparison with just local estimation and investigate the tradeoff with the additional communication load. 

**Figure 16:**
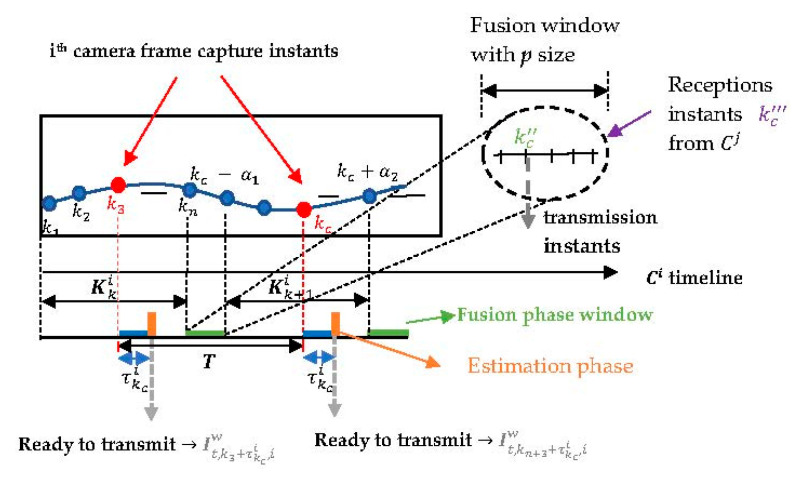
After each time window, the algorithm enters the fusion phase, where information is exchanged asynchronously within a fusion window with *p* size. $k_{c}^{\prime \prime }$ is the transmission instant of $C^{i}$, and $k_{c}^{\prime \prime \prime }$ represents the reception instants in $C^{i}$ from neighboring $C^{j}$ within the fusion window. Content exchanged within the fusion window: information pair (${\tilde{y}}_{t,k_{c}^{\prime \prime },3D}^{i,i},{\tilde{Y}}_{t,k_{c}^{\prime \prime },3D}^{i,i}$) in BAF, fused 3D position measurement, and covariance ($z_{t,k_{c},3D}^{i}$,$\;C_{t,k_{c},3D}^{i}$) in SA-PF.

For that purpose, SA-PF and BAF have their algorithms modified as follows. After the local estimation at the *i*-th camera used the fused 3D position measurement from the asynchronous *LoS* vectors received within the time window $K_{k}^{i}$, further fusion occurs with the additional content received asynchronously from the neighboring cameras within a fusion window with a configurable *p* size. This additional content being exchanged is either the information pair (${\tilde{y}}_{t,k_{c}^{\prime \prime },3D}^{i,i},{\tilde{Y}}_{t,k_{c}^{\prime \prime },3D}^{i,i}$) in BAF or the fused 3D position measurement and covariance ($z_{t,k_{c},3D}^{i}$, $C_{t,k_{c},3D}^{i}$) in SA-PF. Figure 16 represents the actions occurring in two time windows in sequence. The motivation to use SA-PF is that it is not limited to the linear Gaussian assumption of the information filter.

We then investigate whether an improvement occurs with the additional communication exchange in the fusion window. Define $k_{c}^{\prime \prime }$ and $k_{c}^{\prime \prime \prime }$ as, respectively, transmit and reception instants when camera $C^{i}$ exchanges with neighboring cameras $C^{j}$ either the information pair in BAF or the fused 3D position measurement and covariance in SA-PF. Fusion with additional communication exchange in BAF and SA-PF is as follows:

(1) BAF fusion: The *t*-th target information pair is propagated from frame capture instant $k_{c}$ to $k_{c}^{\prime \prime }$, thus yielding (${\tilde{y}}_{t,k_{c}^{\prime \prime },3D}^{i,i},{\tilde{Y}}_{t,k_{c}^{\prime \prime },3D}^{i,i}$) for $C^{i}$ transmission. Define $k_{c}^{\prime \prime \prime }$ to be the reception instant at which camera $C^{i}$ receives an information pair estimate (${\tilde{y}}_{t,k_{c}^{\prime \prime \prime },3D}^{i,j},{\tilde{Y}}_{t,k_{c}^{\prime \prime \prime },3D}^{i,j}$) in the fusion window. We use the Euclidean distance applied to the corresponding 3D centroid position to indicate whether the target is the same. Then, all the received information pairs’ estimates are propagated backward in time to $k_{c}$ for Kullback–Leibler Average (KLA) fusion with the local *i*-th camera information pair estimate (${\tilde{y}}_{t,k_{c},3D}^{i,i},{\tilde{Y}}_{t,k_{c},3D}^{i,i}$), and the result propagated to the next frame capture instant $k_{c}+1$.
(2) SA-PF fusion: As stated before, $C^{i}$ sends the fused 3D measurement $z_{t,k_{c},3D}^{i}$ and covariance $C_{t,k_{c},3D}^{i}$ at instant $k_{c}^{\prime \prime }$ to the neighboring cameras $C^{j}$ without time propagation from $k_{c}$ to $k_{c}^{\prime \prime }$. At various instants $k_{c}^{\prime \prime \prime }$, $C^{i}$ receives ($z_{t,k_{c}^{\prime \prime \prime },3D}^{j}$,$C_{t,k_{c}^{\prime \prime \prime },3D}^{j}$) from neighboring cameras, only once from each $C^{j}$. Here as well we use Euclidean distance applied to 3D position measurement to indicate whether the target is the same. Fusion occurs in this filter by propagating particles’ state estimates from the capture instant $k_{c}$ to the distinct receiving instants $k_{c}^{\prime \prime \prime }$ and sequentially updates the respective weights with ($z_{t,k_{c}^{\prime \prime \prime },3D}^{j}$,$C_{t,k_{c}^{\prime \prime \prime },3D}^{i}$). At the end of the fusion window, $C^{i}$ updates the state estimate at $k_{c}$ and propagates to the next frame capture instant $k_{c}+1$.

## 3. Asynchronous Bayesian State Estimation

Figure 16 shows how we changed the timeline of the Batch Asynchronous Filter (BAF)-Lower Load Mode (LLM) [15] and the Sequential Asynchronous Particle Filter (SA-PF) [17] to accommodate further fusion. Our purpose here is to investigate whether improved target tracking across the asynchronous camera network results from further communication other than just exchanging *LoS* vectors among neighboring cameras to compute fused 3D position measurements and then use local estimation. 

Neighboring cameras transmit either the information pairs’ estimates (BAF) or the fused 3D position measurement and covariance (SA-PF) at instant $k_{c}^{\prime \prime }$ in the end of the time window $K_{k}^{i}$**,** and introduce a fusion window with configurable *p* duration after $K_{k}^{i}$. At instant $k_{c}^{\prime \prime \prime }$ within the fusion window, neighboring cameras receive asynchronously either information pairs’ estimates (BAF) or fused 3D position measurements and covariances (SA-PF). 

Relative to local estimation with fused 3D position measurements, we investigate (a) the KLA-based fusion of received information pairs’ estimates temporally aligned in batch with the local frame capture instant $k_{c}$ (BAF), or (b) the sequential update of particles’ weights by sampling from a likelihood density function with the received fused 3D position measurements and covariances (SA-PF). 

The following Algorithm 1 describes the steps to investigate the effect of additional communications and fusion other than exchanging *LoS* vectors among neighboring cameras for computing fused 3D position measurements:
**Algorithm1.** Algorithm to investigate the effect on target tracking of further fusion other than exchanged LoS vectors and local estimation**Input**: I–images taken by camera $C^{i}$ **Output**: estimated state $x_{t,k_{c},3D}^{i}$ and the root mean square error computation $\epsilon_{t,k_{c},2D}^{i}$
**for** r Monte-Carlo simulation runs **for** each camera 1-If *t*-th target detection is confirmed after three occurrences in images, its position estimate $\;{\hat{z}}_{t,k_{c},3D}^{i}$ is computed as follows: **for** each detected target 2-Detect target centroids in image plane $z_{t,k,2D}^{i}$ within the time window period $K_{k}^{i}$; 3-Transform measurements $z_{t,k,2D}^{i}$ into stored information vectors $i_{t,k,i}^{w}=\{k;O_{c,i}^{w};{\vec{LoS}}_{t,k,i}^{w}\}$; 4-Asynchronous transmission/reception of information vectors $I_{t,k_{c}+\tau_{k_{c}}^{i},i}^{w}=\{k_{c}+\tau_{k_{c}}^{i};O_{c,i}^{w};{\vec{LoS}}_{t,k_{c}+\tau_{k_{c}},i}^{w}\}$ among cameras $C^{i}$ and $C^{j}$, $j\in N^{j,i},\;i\neq j$; 5-Compute 3D position measurements $z_{t,k,3D}^{i}$ and covariances $C_{t,k,3D}^{i}$ within the time window $K_{k}^{i}$ from received *LoS* vectors, and interpolate all of them into temporal alignment with the frame capture instant $k_{c}$ to account for frame processing delay and camera asynchrony; 6-Local estimation of the target position and velocity in 3D space $x_{t,k_{c},3D}^{i}$; 7-Investigate the added fusion phase with either the *BAF* or the *SA-PF* Asynchronously transmit/receive information pairs’s estimates (BAF) or fused 3D position measurements (SA-PF) among cameras $C^{i}$ and $C^{j}$, $j\in N^{j,i},\;j\neq i$; 8-${\hat{z}}_{t,k_{c},3D}^{i}$ is the resulting 3D position component of the target state estimate; 9-Perspective projection of the 3D position estimate of each target being tracked onto the 2D image planes of each neighboring camera, expressed in pixel coordinates: ${\hat{z}}_{t,k_{c},3D}^{i}\to {\hat{z}}_{t,k_{c},2D}^{i}$; end **for**; end **for**; 10-Average root mean square error $\epsilon_{t,k_{c},2D}^{i}=\sqrt{∥{\hat{z}}_{t,k_{c},2D,r}^{i}-z_{t,k_{c},2D}^{i}{∥}_{2}^{2}}$ to compare performance in steps 6 and 7. **end for.**

### 3.1. Batch Asynchronous Filter (BAF)-Lower Load Mode (LLM) [15]

The initialization of each target involves the fused 3D position measurement $z_{t,k_{c},3D}^{i}$ and respective covariance $C_{t,k_{c},3D}^{i}$. Each camera $C^{i}$ uses Kalman filtering in information form [7]. The fused 3D position measurement pair $\left(z_{t,k_{c},3D}^{i},C_{t,k_{c},3D}^{i}\right)$ is transformed in the measurement information pair $\left(H^{T}C_{t,k_{c},3D}^{i}{}^{-1}z_{t,k_{c},3D}^{i},H^{T}C_{t,k_{c},3D}^{i}{}^{-1}H\;\right)$. 

Local estimation uses the measurement information pair and the information pair (${\hat{y}}_{t,k_{c},3D}^{i}$,${\hat{Y}}_{t,k_{c},3D}^{i}$) predicted from the previous frame capture instant to update locally the information pair to $\left(y_{t,k_{c,},3D}^{i,i},Y_{t,k_{c},3D}^{i,i}\right)$ of the *t*-th target at camera $C^{i}$ where $y_{t,k_{c,},3D}^{i,i}={\hat{y}}_{t,k_{c},3D}^{i}+H^{T}C_{t,k_{c},3D}^{i}{}^{-1}z_{t,k_{c},3D}^{i}$ and $Y_{t,k_{c},3D}^{i,i}={\hat{Y}}_{t,k_{c},3D}^{i}+H^{T}C_{t,k_{c},3D}^{i}{}^{-1}H$. Then, ${\hat{z}}_{t,k_{c},3D}^{i}=H{\left(Y_{t,k_{c},3D}^{i,i}\right)}^{-1}y_{t,k_{c,},3D}^{i,i}$ is the 3D position estimate of the target for later use to evaluate performance in step 6 of the Algorithm 1 with the average root mean square error in the image plane. 

To investigate the added fusion phase in step 7 of the Algorithm 1, for the purpose of transmission $C^{i}$ predicts the information pair at instant $k_{c}^{\prime \prime }$ within the fusion window illustrated in Figure 16 as follows:

(14) $$\begin{array}{c} {\tilde{Y}}_{t,k_{c}^{\prime \prime },3D}^{i,i}={\left(F\left(k_{c},k_{c}^{\prime \prime }\right)Y_{t,k_{c},3D}^{i,i}{}^{-1}F{\left(k_{c},k_{c}^{\prime \prime }\right)}^{T}+Q\left(k_{c},k_{c}^{\prime \prime }\right)\right)}^{-1}\\ {\tilde{y}}_{t,k_{c}^{\prime \prime },3D}^{i,i}={\tilde{Y}}_{t,k_{c}^{\prime \prime },3D}^{i,i}\;F\left(k_{c},k_{c}^{\prime \prime }\right)\left(Y_{t,k_{c},3D}^{i,i}{}^{-1}y_{t,k_{c},3D}^{i,i}\right). \end{array}$$

$C^{i}$ transmits the predicted information pair $\left({\tilde{y}}_{t,k_{c}^{\prime \prime },3D}^{i,i},{\tilde{Y}}_{t,k_{c}^{\prime \prime },3D}^{i,i}\right)$ to all cameras $j\in N^{j,i}$. Furthermore, $C^{i}$ receives at the local instant $k_{c}^{\prime \prime \prime }$ within the fusion window the information pair $\left(y_{t,k_{c}^{\prime \prime },3D}^{j,j},Y_{t,k_{c}^{\prime \prime },3D}^{j,j}\right)$ from a neighboring camera $C^{j}$, as depicted in Figure 16. Camera $C^{i}$ has no knowledge of the frame capture instant of neighboring camera $C^{j}$. Therefore, $C^{i}$ reads the information pair received from $C^{j}$ as $\left(y_{t,k_{c}^{\prime \prime \prime },3D}^{i,j},Y_{t,k_{c}^{\prime \prime \prime },3D}^{i,j}\right)$, which undergoes propagation backward in time to the local frame capture instant $k_{c}$:

(15) $$\begin{array}{c} {\tilde{Y}}_{t,k_{c}\;,3D}^{i,j}={\left(F\left(k_{c}^{\prime \prime \prime },k_{c}\;\right)Y_{t,k_{c}^{\prime \prime \prime },3D}^{i,j}{}^{-1}F{\left(k_{c}^{\prime \prime \prime },k_{c}\;\right)}^{T}+Q\left(k_{c}^{\prime \prime \prime },k_{c}\;\right)\right)}^{-1}\\ {\tilde{y}}_{t,k_{c}\;,3D}^{i,j}={\tilde{Y}}_{t,k_{c}\;,3D}^{i,j}\;F\left(k_{c}^{\prime \prime \prime },k_{c}\;\right)\left(Y_{t,k_{c}^{\prime \prime \prime },3D}^{i,j}{}^{-1}y_{t,k_{c}^{\prime \prime \prime },3D}^{i,j}\right). \end{array}$$

Fusion of local and received information pairs at camera $C^{i}$ for each target at instant $k_{c}$ is obtained with the Kullback–Leibler Average (KLA) [20] as follows:

(16) $$\begin{array}{c} {\hat{Y}}_{t,k_{c},3D}^{i}=\frac{1}{n\left(N^{j,i}\right)+1}\left(Y_{t,k_{c},3D}^{i,i}+\overset{n\left(N^{j,i}\right)}{\underset{j,j\in N^{j,i}}{\sum }}{\tilde{Y}}_{t,k_{c},3D}^{i,j}\right)\\ {\hat{y}}_{t,k_{c},3D}^{i}=\frac{1}{n\left(N^{j,i}\right)+1}\left(y_{t,k_{c},3D}^{i,i}+\overset{n\left(N^{j,i}\right)}{\underset{j,j\in N^{j,i}}{\sum }}{\tilde{y}}_{t,k_{c},3D}^{i,j}\right). \end{array}$$

where $n\left(N^{j,i}\right)$ indicates the number of neighboring cameras in the set $N^{j,i}$ whose information pairs of the *t*-th target are received within the fusion window of the *i*-th camera. Then, ${\overset{˘}{z}}_{t,k_{c},3D}^{i}=H{\left({\hat{Y}}_{t,k_{c},3D}^{i}\right)}^{-1}{\hat{y}}_{t,k_{c},3D}^{i}$ is the fused 3D position estimate of the target in step 7 of the Algorithm 1 for later use to evaluate performance with the average root mean square error in the image plane.

We define as strategy (a) the local estimation in step 6, with exchange of just the information vectors $I_{t,k_{c}+\tau_{k_{c}}^{i},i}^{w}$ conveying *LoS* vectors. This strategy predicts from the updated ($y_{t,k_{c,},3D}^{i,i},Y_{t,k_{c},3D}^{i,i}$) to $\left({\hat{y}}_{t,k_{c}+1,3D}^{i},\;{\hat{Y}}_{t,k_{c}+1,3D}^{i}\right)$ in the next capture instant $k_{c}+1$, and computes the next measurement information pair $\left(H^{T}C_{t,k_{c}+1,3D}^{i}{}^{-1}z_{t,k_{c}+1,3D}^{i},H^{T}C_{t,k_{c}+1,3D}^{i}{}^{-1}H\right)$ based on the fused 3D position measurement and covariance, which we compute with *LoS* vectors received in the next time window $K_{k+1}^{i}$.

**Figure 17:**
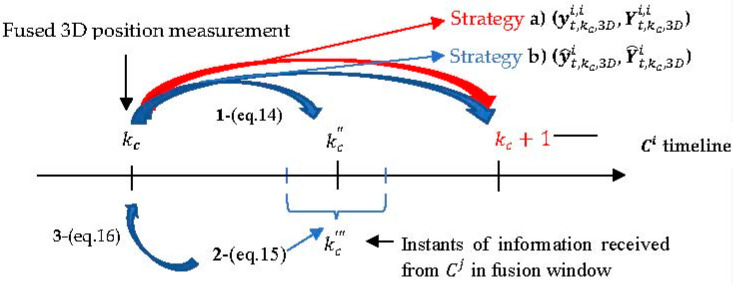
In strategy (a) only the fused 3D position measurement and local estimation to the next time window $K_{k+1}^{i}$ are involved. Three steps are shown for strategy (b) where the blue arrows indicate the actions the filter goes through. The fused 3D measurement $z_{t,k_{c},3D}^{i}$ and covariance $C_{t,k_{c},3D}^{i}$ correspond to the frame capture instant $k_{c}$.

For comparison, we define strategy (b) in step 7 as adding KLA-based fusion with information pairs’ estimates received from neighboring cameras within the fusion window other than the *LoS* vectors received within $K_{k}^{i}$. This strategy predicts from the KLA-fused information pair (${\hat{y}}_{t,k_{c},3D}^{i},{\hat{Y}}_{t,k_{c},3D}^{i}$) to $\left({\hat{y}}_{t,k_{c}+1,3D}^{i},\;{\hat{Y}}_{t,k_{c}+1,3D}^{i}\right)$ in the next capture instant $k_{c}+1$ and, as in strategy (a), computes the next measurement information pair $\left(\;H^{T}C_{t,k_{c}+1,3D}^{i}{}^{-1}z_{t,k_{c}+1,3D}^{i},H^{T}C_{t,k_{c}+1,3D}^{i}{}^{-1}H\;\right)$ from the fused 3D position measurement and covariance from *LoS* vectors received in the next time window $K_{k+1}^{i}$. For clarification, see Figure 17.

The position estimate of the *t*-th target resulting from strategies (a) and (b) at camera $C^{i}$ at frame capture instant $k_{c}$ are, respectively, ${\hat{z}}_{t,k_{c},3D\;}^{i}=HY_{t,k_{c},3D}^{i,i}{}^{-1}y_{t,k_{c,},3D}^{i,i}$ and ${\overset{ˇ}{z}}_{t,k_{c},3D\;}^{i}=H\;{\hat{Y}}_{t,k_{c},3D}^{i}{}^{-1}{\hat{y}}_{t,k_{c},3D}^{i}$. 

### 3.2. Sequential Asynchronous Particle Filter [17]

The initial step for obtaining a fused 3D position measurement pair $\left(z_{t,k_{c},3D}^{i},C_{t,k_{c},3D}^{i}\right)$ at each camera $C^{i}$ is the same as previously described. Each camera has the same inter-frame period $\;T=\alpha_{1}+\alpha_{2}$ between consecutive frame capture instants as depicted in Figure 10. The model of the *t*-th target is the same from Section 3.2.

Define $k_{c}^{\prime }$ at the previous frame capture instant ($k_{c}^{\prime }=k_{c}-1)$ and $m=1,\ldots ,n\left(N^{j,i}\right)$, where we recall that $n\left(N^{j,i}\right)$ indicates the number of neighboring cameras $C^{j},\;j\in N^{j,i}$, whose fused 3D position measurements $z_{t,k_{c}^{\prime \prime \prime },3D}^{j_{m}}$ of the *t*-th target are received within the fusion window of the *i*-th camera. Each camera $C^{i}$ receives measurements $z_{t,k_{c}^{\prime \prime \prime },3D}^{j_{m}}$ at distinct instants $k_{c}^{\prime \prime \prime }$ within the fusion window and computes the corresponding state estimates ${\tilde{x}}_{t,k_{c}^{\prime \prime \prime },3D}^{i,j_{m}}$. The updated state estimate in $C^{i}$ is then computed from ${\tilde{x}}_{t,k_{c}^{\prime \prime \prime },3D}^{i,j_{m}}$, $j_{m}=\left\{j_{1},j_{2},\ldots ,j_{n\left(N^{j,i}\right)}\right\}$, and $j_{0}$ is the $C^{i}$ camera. As an example, let camera 2 (i=2) receive measurements from $n\left(N^{j,i}\right)=3$ neighboring cameras, say, cameras 5, 7, and 9; then m=1,2,3, $N^{j,2}=\left\{j_{1},j_{2},j_{3}\right\}=\left\{5,7,9\right\}$, and $j_{0}=2$.

In the prediction process of SA-PF, new particles $x_{t,k_{c},3D}^{i,\left(l\right)}$ sample the state space from a proposal pdf $q\left(x_{t,k_{c},3D}^{i,\left(l\right)}|x_{t,k_{c}^{\prime },3D}^{i,\left(l\right)},z_{t,k_{c},3D}^{i}\right)$ with the set of previous particles $x_{t,k_{c}^{\prime },3D}^{i,\left(l\right)}$ and weights $\omega_{t,k_{c}^{\prime }}^{i,\left(l\right)}$, and a normalization yields weights ${\hat{\omega }}_{t,k_{c}}^{i,\left(l\right)}$ associated with particles $x_{t,k_{c},3D}^{i,\left(l\right)}$. Then follows the update process; when $C^{i}$ asynchronously receives a measurement $z_{t,k_{c}^{\prime \prime \prime },3D}^{j_{m}}$ from camera $C^{j_{m}}$ in the fusion window, the predicted particles ${\tilde{x}}_{t,k_{c}^{\prime \prime \prime },3D}^{i,j_{m},\left(l\right)}$ are computed, and the corresponding weights ${\hat{\omega }}_{t,k_{c}^{\prime \prime \prime }}^{i,j_{m},\left(l\right)}$ are updated sequentially using measurements $z_{t,k_{c}^{\prime \prime \prime },3D}^{j_{m}}$ and particles ${\tilde{x}}_{t,k_{c}^{\prime \prime \prime },3D}^{i,j_{m},\left(l\right)}$ in the likelihood function. After receiving the measurement from the last of the neighboring cameras within the fusion window, the updated state estimate $x_{t,k_{c},3D}^{i}$ is then computed with the set of weights $\omega_{t,k_{c}}^{i,\left(l\right)}$ and particles $x_{t,k_{c},3D}^{i,\left(l\right)}$.

Prediction. At $k_{c}=0$, L particles initialize with $x_{t,0,3D}^{i,\left(l\right)},l=1,\ldots ,L,$ sampled from the known *pdf* $p\left(x_{t,0,3D}^{i}\right)$. The weights are initialized equally as $\omega_{t,0}^{i,\left(l\right)}=1/L$ for all l.

At instants $k_{c}\geq 1$ with $z_{t,k_{c},3D}^{i}$ previously obtained, new particles $x_{t,k_{c},3D}^{i,\left(l\right)}$ are propagated from the previous particles $x_{t,k_{c}^{\prime },3D}^{i,\left(l\right)}$ with the deterministic part of the motion model in Equation (10). The corresponding weights ${\tilde{\omega }}_{t,k_{c}}^{i,\left(l\right)}$ use the fused 3D position measurement $z_{t,k_{c},3D}^{i}$:

(17) $${\tilde{\omega }}_{t,k_{c}}^{i,\left(l\right)}=\omega_{t,k_{c}^{\prime }}^{i,\left(l\right)}\frac{p(z_{t,k_{c},3D}^{i}|x_{t,k_{c},3D}^{i,\left(l\right)})\;p(x_{t,k_{c},3D}^{i,\left(l\right)}|x_{t,k_{c}^{\prime },3D}^{i,\left(l\right)})}{q\left(x_{t,k_{c},3D}^{i,\left(l\right)}|x_{t,k_{c}^{\prime },3D}^{i,\left(l\right)},z_{t,k_{c},3D}^{i}\right)}=\omega_{t,k_{c}^{\prime }}^{i,\left(l\right)}p(z_{t,k_{c},3D}^{i}|x_{t,k_{c},3D}^{i,\left(l\right)})$$

The above weights are normalized according to ${\hat{\omega }}_{t,k_{c}}^{i,\left(l\right)}={\tilde{\omega }}_{t,k_{c}}^{i,\left(l\right)}/\overset{L}{\underset{l^{\prime }=1}{\sum }}{\tilde{\omega }}_{t,k_{c}}^{i,\left(l^{\prime }\right)}$. The set of particles and respective normalized weights is ${\left\{x_{t,k_{c},3D}^{i,\left(l\right)},{\hat{\omega }}_{t,k_{c}}^{i,\left(l\right)}\right\}}_{l=1}^{L}$. This is the local estimation defined as strategy (a), wherein SA-PF becomes just PF, which does not involve exchange other than *LoS* vectors, ${\hat{x}}_{t,k_{c},3D}^{i}=\overset{L}{\underset{l=1}{\sum }}{\hat{\omega }}_{t,k_{c}}^{i,\left(l\right)}x_{t,k_{c},3D}^{i,\left(l\right)}$, and follows the propagation to the next frame capture instant $k_{c}+1$ (step 6 of the pseudoalgorithm).

Update. Strategy (b) or step 7 of the Algorithm 1 involves the fusion window, when $C^{i}$ receives asynchronously measurements $z_{t,k_{c}^{\prime \prime \prime },3D}^{j_{m}}$ from the neighboring cameras $C^{j}$ *at distinct reception instants* $k_{c}^{\prime \prime \prime }$, then SA-PF predicts particles ${\tilde{x}}_{t,k_{c}^{\prime \prime \prime },3D}^{i,j_{m},\left(l\right)}$ and updates the weights ${\hat{\omega }}_{t,k_{c}^{\prime \prime \prime }}^{i,\left(l\right)}$ as in Equations (18) and (19) respectively.

Initially, ${\hat{\omega }}_{t,k_{c}^{\prime \prime \prime }}^{i,j_{0},\left(l\right)}={\hat{\omega }}_{t,k_{c}}^{i,\left(l\right)}$ described above and the predicted particles ${\tilde{x}}_{t,k_{c}^{\prime \prime \prime },3D}^{i,j_{m},\left(l\right)}$ are:

(18) $${\tilde{x}}_{t,k_{c}^{\prime \prime \prime },3D}^{i,j_{m},\left(l\right)}=E\left\{x_{t,k_{c}^{\prime \prime \prime },3D}^{j_{m},\left(l\right)}|x_{t,k_{c},3D}^{i,\left(l\right)}\right\}=F\left(k_{c},k_{c}^{\prime \prime \prime }\right)x_{t,k_{c},3D}^{i,\left(l\right)}$$

The corresponding weights ${\hat{\omega }}_{t,k_{c}^{\prime \prime \prime }}^{i,j_{m},\left(l\right)},l=1,\ldots ,L$ are updated sequentially according to:

(19) $${\tilde{\omega }}_{t,k_{c}^{\prime \prime \prime }}^{i,j_{m},\left(l\right)}={\hat{\omega }}_{t,k_{c}^{\prime \prime \prime }}^{i,j_{m-1},\left(l\right)}p\left(z_{t,k_{c}^{\prime \prime \prime },3D}^{j_{m}}|{\tilde{x}}_{t,k_{c}^{\prime \prime \prime },3D}^{i,j_{m},\left(l\right)}\right),\;\;m=1,\ldots ,n\left(N^{j,i}\right)\;\;$$

where $p(z_{t,k_{c}^{\prime \prime \prime },3D}^{j_{m}}|{\tilde{x}}_{t,k_{c}^{\prime \prime \prime },3D}^{i,j_{m},\left(l\right)})$ is the likelihood function in Equation (21). The weights in Equation (19) are normalized according to ${\hat{\omega }}_{t,k_{c}^{\prime \prime \prime }}^{i,j_{m},\left(l\right)}={\tilde{\omega }}_{t,k_{c}^{\prime \prime \prime }}^{i,j_{m},\left(l\right)}/\overset{L}{\underset{l^{\prime }=1}{\sum }}{\tilde{\omega }}_{t,k_{c}^{\prime \prime \prime }}^{i,j_{m},\left(l^{\prime }\right)}$. The set of particles and normalized weights ${\left\{{\tilde{x}}_{t,k_{c}^{\prime \prime \prime },3D}^{i,j_{m},\left(l\right)},{\hat{\omega }}_{t,k_{c}^{\prime \prime \prime }}^{i,j_{m},\left(l\right)}\right\}}_{l=1}^{L}$ represent the “partial posterior” $p\left({\tilde{x}}_{t,k_{c},3D}^{i\left(j_{0}:j_{m}\right)}|z_{t,k_{c},3D}^{i\left(j_{0}:j_{m}\right)},z_{t,1:k_{c}^{\prime },3D}\right)$. This conditioning is on all previous measurements $z_{t,1:k_{c}^{\prime },3D}$, and all current measurements $z_{t,k_{c},3D}^{i\left(j_{0}:j_{m}\right)}$ received at camera $C^{i}$ during the fusion window at distinct instants $k_{c}^{\prime \prime \prime }$, including the local $z_{t,k_{c},3D}^{i}$ and up to the neighboring camera $C^{j_{m}}$ as indicated by superscript $\left(j_{0}:j_{m}\right)$ [17].

This update in SA-PF is sequential until the last remaining measurement from the neighboring cameras is received within the fusion window, and then the “global posterior” weights [17] become $\omega_{t,k_{c}}^{i,\left(l\right)}={\hat{\omega }}_{t,k_{c}^{\prime \prime \prime }}^{i,j_{n\left(N^{j,i}\right)},\left(l\right)}$. The set of particles and respective normalized weights is ${\left\{x_{t,k_{c},3D}^{i,\left(l\right)},\omega_{t,k_{c}}^{i,\left(l\right)}\right\}}_{l=1}^{L}$, and the state estimate is $x_{t,k_{c},3D}^{i}=\overset{L}{\underset{l=1}{\sum }}\omega_{t,k_{c}}^{i,\left(l\right)}x_{t,k_{c},3D}^{i,\left(l\right)}$. Finally, we propagate the particles $x_{t,k_{c},3D}^{i,\left(l\right)}$ to the next frame capture instant $k_{c}+1$ with the deterministic part of the motion model in Equation (10).

## 4. Simulation Results

### 4.1. Evaluation Setup

**Figure 18:**
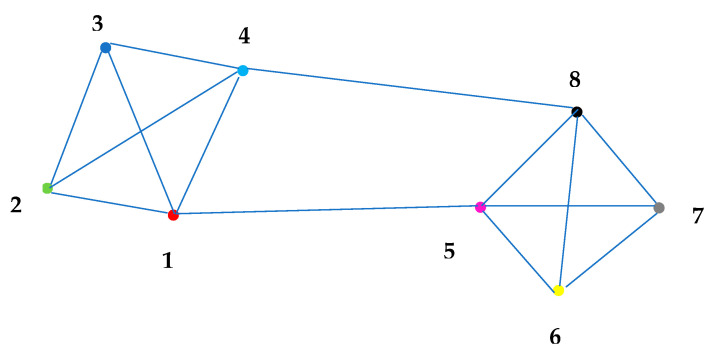

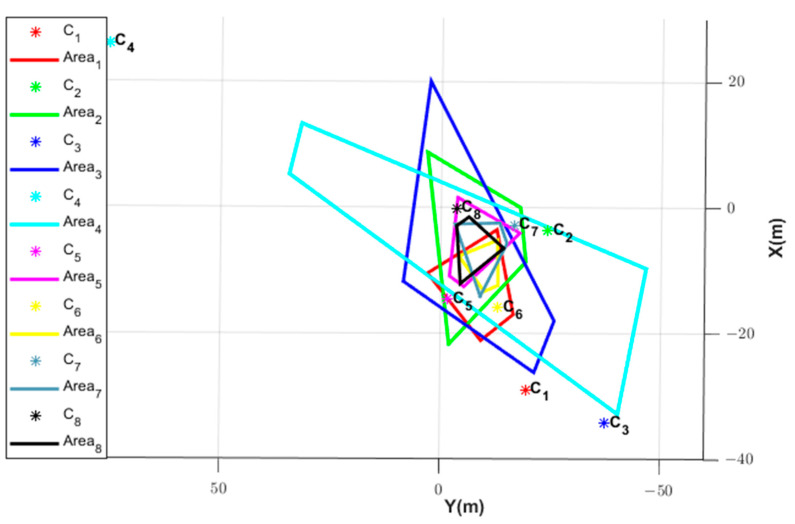
Top: network communications topology. Bottom: projection of the *FoVs* from each of the $N_{s}=8$ cameras on the ground reference plane.

We use MATLAB [42] and the PETS2009 database [27] with a camera network composed of $N_{s}=8$ cameras. The targets move within the surveillance volume of a network of calibrated cameras, whose construction (intrinsic) parameters and poses are given in the header of the PETS2009 image sequence dataset file. This dataset is challenging for tracking tasks because targets change direction, and clusters and scattering occur frequently. Scene illumination and obstacles also hinder tracking. In this experiment, the frame sampling rate was 7 frames/s ≈143 ms, with a total of 794 frames per camera. Each k time step takes approximately 143 ms. Figure 18 shows all *FoVs* projected on the ground reference plane for each of the eight cameras. Portions of some *FoVs* do not project on the ground and instead cover the scenario above ground. The surveillance area of the cameras is approximately 60 m × 90 m as depicted in Figure 18, and their *FoVs* overlap. Moreover, the neighborhoods for each camera are defined by the network communication topology also shown. The chosen topology reflects two camera neighborhoods (clusters), one with cameras having large *FoVs* areas projected on the ground and the other one consisting of cameras with smaller projections. Communications between the clusters occur by means of cameras 4 and 8 and cameras 1 and 5.

BAF assumes the information *pdf*s are Gaussian whereas PF makes no such assumption. They are evaluated under the two strategies (a) and (b) previously explained.

The random process modeling the processing delay in frame units is $\tau_{k}^{i}\in U\left\{0,\tau^{max}\right\}$, where U is the uniform distribution. A uniform distribution is also used for the offset $\alpha^{ij}$ between cameras: $U\left\{0,\alpha^{max}\right\}$. Denote $\Lambda^{i,r}\subset \left[1,749\right]$ as the set of frame capture instants during the *r*-th run of a Monte Carlo simulation, *r* = 1, …, *M* of camera $C^{i}$ tracking a target and $P_{k}^{i}\subset \left\{1,\ldots ,N_{t}\right\}$ as the set of targets in the *FoV* of camera $C^{i}$ at capture instant $k_{c}$. The evaluation is based on the average root mean square error:

(20) $$\varepsilon =\sqrt{\frac{1}{MN_{c}}\overset{M}{\underset{r=1}{\sum }}\overset{N_{c}}{\underset{i=1}{\sum }}\frac{1}{\left|\Lambda^{i,r}\right|}\underset{\forall k\in \Lambda^{i,r}}{\sum }\frac{1}{\left|{Ρ}_{k}^{i}\right|}\underset{\forall t\in P_{k}^{i}}{\sum }||{\hat{z}}_{t,k_{c},2D,r}^{i}-z_{t,k_{c},2D}^{i}{||}_{2}^{2}}$$

where ${\hat{z}}_{t,k_{c},2D,r}^{i}$ is the estimated 3D position of the *t*-th target projected according to the calibrated pinhole model onto the image plane of camera $C^{i}$ at the instant $k_{c}$ during the *r*-th round, and $z_{t,k_{c},2D}^{i}$ corresponds to the actual measurement in the image plane as provided by the target detection algorithm. The latter does not change with r, since the image sequence dataset is given and processed previously by the target detection algorithm. $\sigma_{u}^{2}=4$ in Equation (12), and $R^{i}=C_{t,k_{c},3D}^{i}$ in the information filter with the measurement information pair $\left(H^{T}C_{t,k_{c},3D}^{i}{}^{-1}z_{t,k_{c},3D}^{i},H^{T}C_{t,k_{c},3D}^{i}{}^{-1}H\;\right)$ and in the likelihood function in Equation (21). F and Q are given by the dynamic model of the target. The error ε statistics converged with M=12 runs.

**Figure 19:**
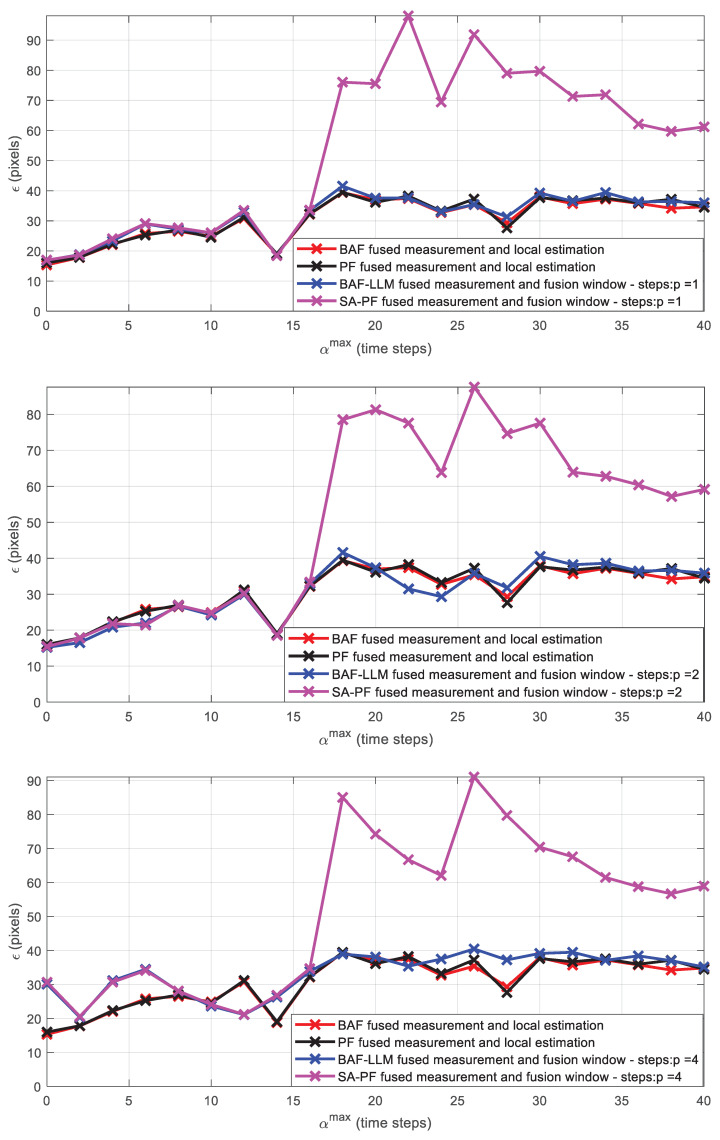

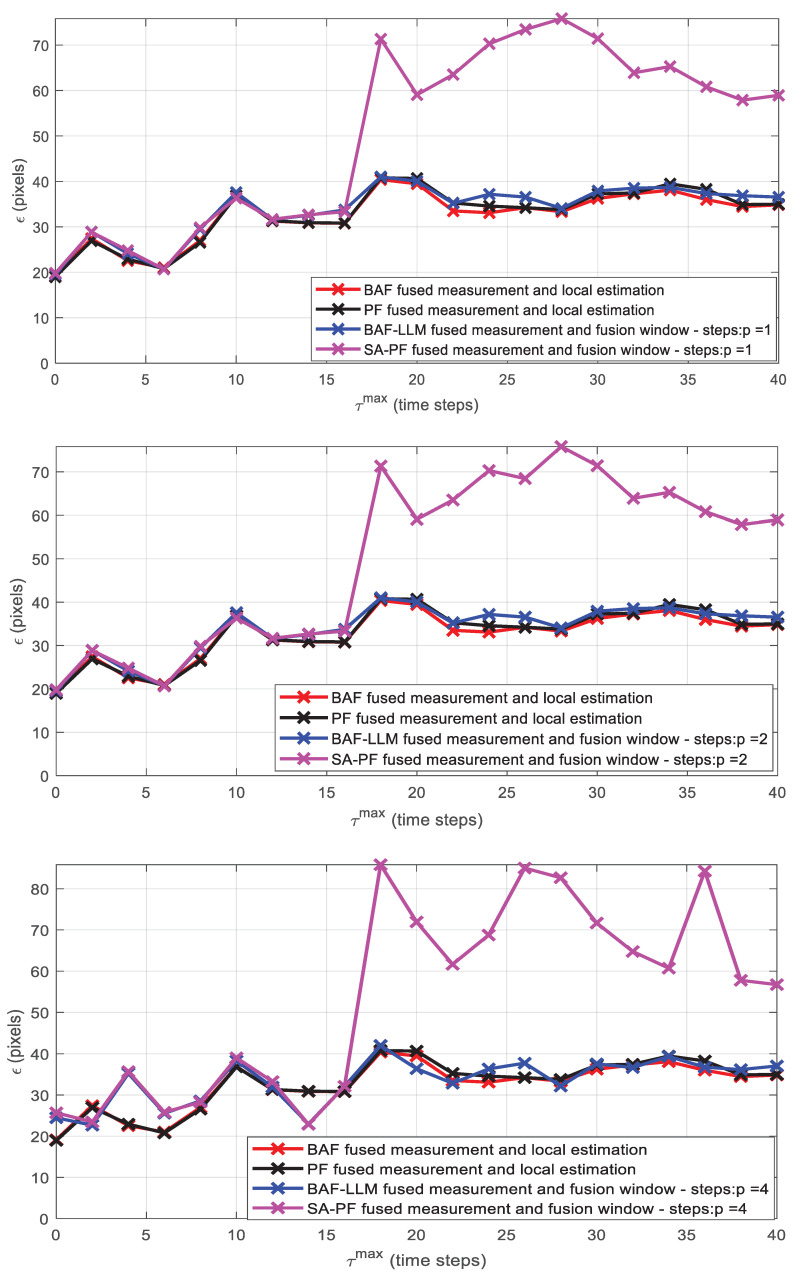
Performance with variation of $\alpha^{max}$ and $\;\tau^{max}$ with exchange of just *LoS* vectors for fused 3D position measurement followed by local estimation (strategy a) and exchange of additional content other than *LoS* vectors for further fusion (strategy b) in fusion window with duration p = {1,2,4} frames.

We skip dataset frames to simulate asynchronous frame capture and frame processing delay. ε is analyzed by increasing the relative offset $0\leq \alpha^{max}\leq 40$ (see Figure 19) with a fixed offset $\alpha^{max}$ and processing delay $\tau_{k}^{i}=\tau^{i}$ at each *i*-th camera and *r*-th run. Each camera has $\tau^{i}$ sampled once from $U\left\{0,\tau^{max}\right\},$ 0 $\leq \tau^{max}\leq$ 3 and remains the same throughout all runs.

The same process is applied to analyze the effect on ε of increasing $0\leq \tau^{max}\leq 40$ (see Figure 19) with a fixed processing delay $\tau^{max}$ and relative offset $\alpha^{i}$ at each *i*-th camera, and *r*-th run. Each camera has $\alpha^{i}$ sampled once from $U\left\{0,\alpha^{max}\right\},$ (synchronous case) $0\leq \alpha^{max}\leq 4\;$ (asynchronous case) and remains the same throughout all runs.

Concerning the particle filter, the likelihood function in Equation (17) is the multivariate Gaussian *pdf* [43]:

(21) $$\begin{array}{c} p\left(z_{t,k_{c},3D}^{i}|x_{t,k_{c},3D}^{i,\left(l\right)}\right)=\\ \frac{1}{{\left(2\pi \right)}^{\frac{N}{2}}det^{\frac{1}{2}}\left(C_{t,k_{c},3D}^{i}\right)}exp\left[-\frac{1}{2}{\left(Hx_{t,k_{c},3D}^{i,\left(l\right)}-z_{t,k_{c},3D}^{i}\right)}^{T}{\left(C_{t,k_{c},3D}^{i}\right)}^{-1}\left(Hx_{t,k_{c},3D}^{i,\left(l\right)}-z_{t,k_{c},3D}^{i}\right)\right], \end{array}$$

N=3 since $z_{t,k_{c},3D}^{i}$ and $C_{t,k_{c},3D}^{i}$ refer to the fused 3D position measurement. Equation (19) $p(z_{t,k_{c},3D}^{j_{m}}|{\tilde{x}}_{t,k_{c}^{\prime \prime \prime },3D}^{i,j_{m},\left(l\right)})\;$ shares the same structure of Equation (21) but changes the corresponding variables. L=1000 particles are used.

Figure 19 shows the simulation results for strategies (a) and (b). (a) exchanges *LoS* vectors among neighboring cameras followed by local estimation using fused 3D position measurements (BAF and PF), and (b) employs a fusion window after the time window $K_{k}^{i}$ with duration p={1,2,4} in frames to process either exchanged information pairs’ estimates (BAF) or fused 3D position measurements (SA-PF) other than *LoS* vectors.

### 4.2. Discussions

Figure 19 shows that BAF and PF filters present similar performance when varying $\alpha^{max}$ and $\tau^{max}$ in all tested situations for strategy (a) where *LoS* vectors measurements exchanged among cameras in the time window $K_{k}^{i}$ generate the fused 3D position measurement $z_{t,k_{c},3D}^{i}$ and both filters use it to estimate the tracked targets’ positions and velocities. The BAF-LLM filter presents a better performance than the SA-PF filter in strategy (b) when $\tau^{max}=\alpha^{max}\geq 16$. The initial weights ${\hat{\omega }}_{t,k_{c}^{\prime \prime \prime }}^{i,j_{0},\left(l\right)}={\hat{\omega }}_{t,k_{c}}^{i,\left(l\right)}$ in the update phase consider the weights ${\hat{\omega }}_{t,k_{c}}^{i,\left(l\right)}$ at instant $k_{c}$ in the receiving instant $k_{c}^{\prime \prime \prime }$, and the instant of the received measurements $z_{t,k_{c}^{\prime \prime \prime },3D}^{j_{m}}$ is considered as the frame capture instant of the neighboring camera, since SA-PF is not informed about the frame capture time at the neighboring camera. Moreover, just one significantly delayed measurement from a neighboring camera yields a small likelihood value, thus compromising the remaining particle weight updates in the sequential fusion step of SA-PF. The small likelihood in such condition is due to the particle state estimate undergoing time propagation in Equation (19) from capture instant to reception instant, whereas the neighboring camera measurement received asynchronously in the fusion window presents a large delay relative to the local camera capture instant. Then, if $k_{c}^{\prime \prime \prime }$ ≫ $k_{c}$, the updated estimate is negatively affected by the delay. Therefore, it is justified that for $\tau^{max}$ and $\alpha^{max}\leq 16$ (small values of processing delay and asynchrony), the SA-PF filter produces an average root mean square error response similar to that of the BAF-LLM filter; i.e., small $\alpha^{max}$ and $\;\tau^{max}$ do not degrade SA-PF filter performance in strategy (b).

As depicted in Figure 19 for all tested *p* = {1,2,4}, the average root mean square error of SA-PF undergoes sudden degradation relative to BAF-LLM when either asynchrony or frame processing delay increase significantly (blue and magenta curves—strategy (b)). BAF-LLM is not susceptible due to its prediction step from the frame capture instant to the transmission instant.

One problem is to validate the target information received by camera $C^{i}$ because, as stated previously, different *Ids* can be assigned to the same target in different cameras. This problem is solved in BAF by evaluating the Euclidean distance between the target 3D position estimate in camera $C^{i}$ with the position estimate received from the neighborhood. In the SA-PF filter, we use the Euclidean distance between measurement $z_{t,k_{c},3D}^{i}$ and measurement $z_{t,k_{c}^{\prime \prime \prime },3D}^{j_{m}}$ received from the neighborhood; i.e., for each information exchange between cameras in the fusion window of a potential target, a distance tolerance ensures the correct fusion of data arising from the same target.

Target tracking by a camera is lost if the camera capture interval $T=\alpha_{1}+\alpha_{2}$ exceeds the target duration within the camera *FoV*. Different from [15] where a fixed number of targets remain within sight of the camera network during the experiment, our distributed target tracking algorithm can track a varying number of targets as they move within the *FoV* of at least 2 cameras.

Cameras in close spatial proximity degrade the 3D estimation accuracy of targets at long range, since the 3D measurement from imaging as proposed here borrow the concept of depth estimation from baseline length and the epipolar constraint in stereoscopy.

Additionally, we conducted further evaluation of (1) the effect of camera pose uncertainty, with focus on camera attitude error, and (2) computational complexity of our proposed 3D position measurement approach.

The PETS2009 image sequence dataset is a real benchmark scenario for target tracking with fixed cameras whose calibration parameters and pose data are accurate. In spite of that, we conduct a Monte Carlo simulation with M = 20 runs wherein uncertainties are added in the extrinsic calibration parameters of each camera (rotation and translation parameters). To the calibrated rotation angles $\theta_{z},\theta_{y},\theta_{x}$ of each camera in radians, we add a Gaussian uncertainty N(μ=0.05 rad,σ=0.001 rad). Additive Gaussian uncertainty N(μ=10 cm,σ=1 cm) in camera location is also applied. We evaluate the mean square error in Equation (20). The simulation investigated processing delays τ = (0, 2, 4, 6, 8, 10, 12, 14, 16, 18), maximum asynchrony among cameras $\alpha_{max}=4$, and the particular asynchrony value for each camera sampled from the uniform distribution $U\left\{0,\alpha^{max}\right\}$, i.e., each camera received $0\leq \;\alpha \leq \alpha_{max}$.

**Figure 20:**
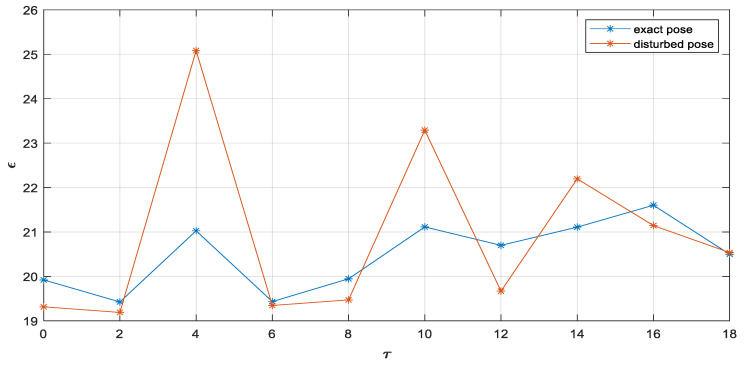
Mean square error performance ϵ in pixels with added uncertainties in cameras’ poses.

Figure 20 indicates uncertainties in cameras’ poses degrade the fused 3D position measurement of targets when compared with exact poses, as expected.

We evaluate numerically the computational complexity by increasing the number $n_{i}$ of targets observed in the field of view of the *i*-th camera and the number $n_{j}$ of targets received by the *i*-th camera of the *j*-th cameras, thus resulting a total of $n_{i}\cdot n_{j}$ targets. The processing tasks considered for each of the targets are:

(a) Variable $n_{i}$—each target has 7 sigma points assigned to the local *LoS* vector pointing to the *t*-th target in the image plane;
(b) Variable $n_{j}$—each target with its 7 sigma points assigned to the received *LoS* vector pointing to the received *t*-th target in the image plane;
(c) Intersection search of pairs of *LoS* from the set of 49 *LoS* vectors providing points in 3D space (Equations (6) and (8));
(d) Checks which 3D points pass the distance criterion d≤0.3 m;
(e) Selects the 7 *LoS* vectors from the set of 49 *LoS* vectors that pass the above test with smallest distance d; and
(f) UT computation: 3D position measurement mean and covariance obtained at a $k_{c}$ capture instant of the *i*-th camera.

**Figure 21:**
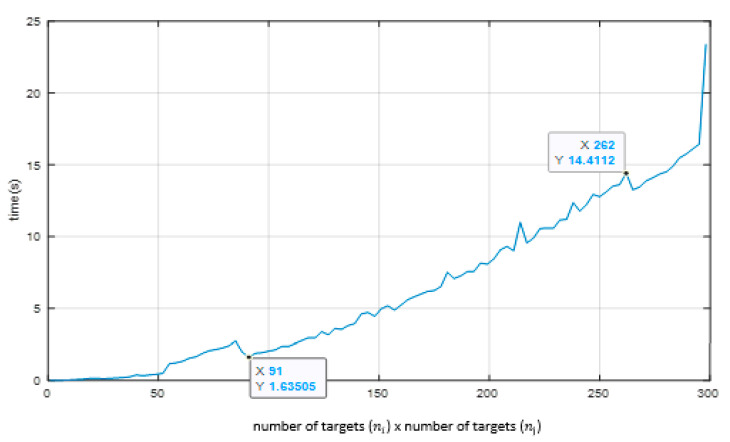
Elapsed time to process tasks (a)–(f) as a function of the total number of targets $n_{i}\cdot n_{j}$.

We noticed three regions in Figure 21:

(a) For the number of targets varying from 1 up to 90 targets (i.e., from 1 × 1 up to 90 × 90 targets), the elapsed processing time shows an exponential tendency;
(b) From 91 up to 262 targets (i.e., from 91 × 91 up to 262 × 262 targets) we observe a linear tendency, and over 262 × 262 targets, the elapsed processing time presents an exponential tendency.

In the PETS2009 image sequence dataset, the number of observed targets at any $k_{c}$ capture instant does not reach above 10 targets simultaneously. Therefore, our approach yields a very low processing time to carry out the 3D position measurements.

## 5. Conclusions

We propose position measurement in 3D space with intersecting vector constraints derived from exchanged *LoS* vectors in a camera network with known relative poses. Solving for the 3D intersection of vector constraints is based on computing the proximity of reverse straight lines. Each camera does not need the construction parameters of the other ones to obtain the 3D measurements. We then applied our approach to tracking simultaneous targets with a network of asynchronously communicating cameras with frame processing delays. The targets enter and leave the fields of view of the cameras in the image sequence dataset.

We use the average root mean square error across all image planes of the asynchronous camera network to measure tracking performance under two conditions: fused 3D position measurement from exchanged *LoS* vectors and local state estimation (strategy (a)), and fusion of additional information other than the exchanged *LoS* vectors (strategy (b)).

BAF and PF present similar performance under strategy (a), which uses local estimation. With regard to strategy (b) involving further fusion, BAF-LLM is superior to SA-PF under large asynchrony and processing delay. Comparing between strategies (a) and (b), BAF, PF, and BAF-LLM yield approximately the same performance in a wide range of asynchrony and processing delay. We remark that local estimation in BAF and PF demands less communications than further fusion used in BAF-LLM and SA-PF. Further investigation should focus on the use of consensus to improve target tracking subject to asynchronous exchange in the camera network.

## Figures and Tables

**Table 1:** Analytical comparison.

Ref.	Acronym	Characteristics				Fusion Method
		Processing Delays	Aligns Received Data to Local Capture Instant	Data Exchanged among Camera Nodes	Position Representation	
[16]	GHF	no	no	State estimates	Polar coordinates	No fusion
[25]	Decentralized EKF	no	no	State estimates	Cartesian coordinates	Sequential
[26]	DAT2TF	no	no	State estimates	Polar coordinates	Sequential
[15]	BAF-LLM	yes	yes	State estimates	Cartesian coordinates	Batch
[17]	SA-PF	no	no	Measurements	Cartesian coordinates	Sequential
This paper [15]	BAF-LLM—local estimation ^1^	yes	yes ^3^	(1) *LoS* vectors to measure 3D positions	Cartesian coordinates	Batch
	BAF-LLM—with further fusion	yes	yes	(2) *LoS* vectors to measure 3D positions and information pairs’ estimates	Cartesian coordinates	Batch
This paper [17]	PF—local estimation ^2^	no	yes ^3^	(1) *LoS* vectors to measure 3D positions	Cartesian coordinates	Batch
	SA-PF—with further fusion	no	no	(2) *LoS* vectors to measure 3D positions and fused 3D position measurements	Cartesian coordinates	Sequential

^1^ Kalman Filter in information form. ^2^ Particle Filter. ^3^ 3D position measurements from asynchronously received *LoS* vectors undergo interpolation to local capture instant for the purpose of time alignment. See Section 2.1.6.

## Data Availability

Not applicable.

## Abbreviations

The following abbreviations are used in this manuscript:

WCN	Wireless Communication Networks
BAF	Batch Asynchronous Filter
BAF-LLM	Batch Asynchronous Filter – Lower Load Mode
SA-PF	Sequential Asynchronous Particle Filter
*LoS*	Line Of Sight
*FoV*	Field Of View

## Appendix A

Minimum point between reversed straight lines:

The expressions describing $\vec{r}$ e $\vec{s}$ are as follows:

$$\{\begin{array}{c} \vec{r}=P_{1}+\lambda {\vec{v}}_{1}\;\\ \vec{s}=P_{2}+\beta {\vec{v}}_{2} \end{array}$$

where λ, β ∈ ${\mathbb{R}}^{+}$ and ${\vec{v}}_{1},{\vec{v}}_{2}$ are the direction vectors. The distances between any two points on lines $\vec{r}$ e $\vec{s}$ can be calculated as follows:

$$d\left(\vec{r},\vec{s}\right)=\sqrt{{\left(r_{x}-s_{x}\right)}^{2}+{\left(r_{y}-s_{y}\right)}^{2}+{\left(r_{z}-s_{z}\right)}^{2}}.$$

To find the minimum point between the lines, it is necessary to find the parameters λ and β that minimize the distance function $d\left(\vec{r},\vec{s}\right)$. Therefore, $\lambda^{*},\beta^{*}=\underset{\lambda ,\beta }{\min}d\left(\lambda ,\beta \right)$ is the partial derivative of the function $d\left(\vec{r},\vec{s}\right)$:

$$\{\begin{array}{c} \frac{\partial d}{\partial \lambda }=0\\ \frac{\partial d}{\partial \beta }=0 \end{array}$$

$$\left(I\right)\frac{\partial d}{\partial \lambda }=0=\frac{1}{2}{\left(d\left(\lambda ,\beta \right)\right)}^{-\frac{1}{2}}\left[2\left(r_{x}-s_{x}\right)\frac{dr_{x}}{d\lambda }+2\left(r_{y}-s_{y}\right)\frac{dr_{y}}{d\lambda }+2\left(r_{z}-s_{z}\right)\frac{dr_{z}}{d\lambda }\right],$$

where

$$\begin{array}{c} \frac{dr_{x}}{d\lambda }=\frac{d\left(p_{1x}+\lambda v_{1x}\right)}{d\lambda }=v_{1x}\\ \frac{dr_{y}}{d\lambda }=\frac{d\left(p_{1y}+\lambda v_{1y}\right)}{d\lambda }=v_{1y}\\ \frac{dr_{z}}{d\lambda }=\frac{d\left(p_{1z}+\lambda v_{1z}\right)}{d\lambda }=v_{1z} \end{array}$$

Then,

$$\left(r_{x}-s_{x}\right)v_{1x}+\left(r_{y}-s_{y}\right)v_{1y}+\left(r_{z}-s_{z}\right)v_{1z}=0$$

$$\left[p_{1x}+\lambda v_{1x}-\left(p_{2x}+\beta v_{2x}\right)\right]v_{1x}+\left[p_{1y}+\lambda v_{1y}-\left(p_{2y}+\beta v_{2y}\right)\right]v_{1y}+\left[p_{1z}+\lambda v_{1z}-\left(p_{2z}+\beta v_{2z}\right)\right]v_{1z}=0$$

Arranging the terms of these equations:

$$\left(p_{1x}-p_{2x}\right)v_{1x}+\left(p_{1y}-p_{2y}\right)v_{1y}+\left(p_{1z}-p_{2z}\right)v_{1z}+\lambda v_{1x}^{2}+\lambda v_{1y}^{2}+\lambda v_{1z}^{2}-\beta \left(v_{1x}v_{2x}+v_{1y}v_{2y}+v_{1z}v_{2z}\right)=0$$

$$(A)\;\left(P_{1}-P_{2}\right).{\vec{v}}_{1}+\lambda ||{\vec{v}}_{1}{||}^{2}-\beta {\vec{v}}_{1}.{\vec{v}}_{2}=0$$

$$\left(II\right)\frac{\partial d}{\partial \beta }=0=\frac{1}{2}{\left(d\left(\lambda ,\beta \right)\right)}^{-\frac{1}{2}}\left[-2\left(r_{x}-s_{x}\right)\frac{ds_{x}}{d\beta }-2\left(r_{y}-s_{y}\right)\frac{ds_{y}}{d\beta }-2\left(r_{z}-s_{z}\right)\frac{ds_{z}}{d\beta }\right]$$

$$\begin{array}{c} \left(r_{x}-s_{x}\right)v_{2x}+\left(r_{y}-s_{y}\right)v_{2y}+\left(r_{z}-s_{z}\right)v_{2z}=0\\ \left(p_{1x}-p_{2x}\right)v_{2x}+\left(p_{1y}-p_{2y}\right)v_{2y}+\left(p_{1z}-p_{2z}\right)v_{2z}-\beta (v_{2x}^{2}+v_{2y}^{2}+v_{2z}^{2})+\lambda \left(v_{1x}v_{2x}+v_{1y}v_{2y}+v_{1z}v_{2z}\right)=0 \end{array}$$

$$(B)\;\left(P_{1}-P_{2}\right).{\vec{v}}_{2}-\beta ||{\vec{v}}_{2}{||}^{2}-\lambda {\vec{v}}_{1}.{\vec{v}}_{2}=0$$

From (A) and (B):

$$\begin{array}{c} \lambda =\frac{\beta {\vec{v}}_{1}.{\vec{v}}_{2}-\left(P_{1}-P_{2}\right).{\vec{v}}_{1}}{||{\vec{v}}_{1}{||}^{2}}\\ \beta =\frac{\left(P_{1}-P_{2}\right).{\vec{v}}_{2}+\lambda {\vec{v}}_{1}.{\vec{v}}_{2}}{||{\vec{v}}_{2}{||}^{2}} \end{array}$$

Substituting β in λ, we finally obtain:

$$\{\begin{array}{c} \lambda^{*}=\frac{\left({\vec{v}}_{1}.{\vec{v}}_{2}\right)\left[\left(P_{1}-P_{2}\right).{\vec{v}}_{2}\right]-||{\vec{v}}_{2}{||}^{2}\left[\left(P_{1}-P_{2}\right).{\vec{v}}_{1}\right]}{||{\vec{v}}_{1}{||}^{2}||{\vec{v}}_{2}{||}^{2}-{\left({\vec{v}}_{1}.{\vec{v}}_{2}\right)}^{2}}\\ \beta^{*}=\frac{\left(P_{1}-P_{2}\right).{\vec{v}}_{2}+\lambda^{*}\left({\vec{v}}_{1}.{\vec{v}}_{2}\right)}{||{\vec{v}}_{2}{||}^{2}} \end{array}$$
